# The *Drosophila Su(var)3–7* Gene Is Required for Oogenesis and Female Fertility, Genetically Interacts with *piwi and aubergine*, but Impacts Only Weakly Transposon Silencing

**DOI:** 10.1371/journal.pone.0096802

**Published:** 2014-05-12

**Authors:** Denis Basquin, Anne Spierer, Flora Begeot, Dmitry E. Koryakov, Anne-Laure Todeschini, Stéphane Ronsseray, Cristina Vieira, Pierre Spierer, Marion Delattre

**Affiliations:** 1 Department of Genetics and Evolution, University of Geneva, Geneva, Switzerland; 2 Institute of Molecular and Cellular Biology SB RAS, Novosibirsk, Russia; 3 Laboratoire Biologie du Développement, UMR7622, CNRS-Université Pierre et Marie Curie, Paris, France; 4 Laboratoire de Biométrie et Biologie Evolutive, UMR5558, Université Lyon1, Villeurbanne, France; 5 Institut Universitaire de France, Paris, France; Georgia Institute of Technology, United States of America

## Abstract

Heterochromatin is made of repetitive sequences, mainly transposable elements (TEs), the regulation of which is critical for genome stability. We have analyzed the role of the heterochromatin-associated Su(var)3–7 protein in *Drosophila* ovaries. We present evidences that Su(var)3–7 is required for correct oogenesis and female fertility. It accumulates in heterochromatic domains of ovarian germline and somatic cells nuclei, where it co-localizes with HP1. Homozygous mutant females display ovaries with frequent degenerating egg-chambers. Absence of Su(var)3–7 in embryos leads to defects in meiosis and first mitotic divisions due to chromatin fragmentation or chromosome loss, showing that Su(var)3–7 is required for genome integrity. Females homozygous for *Su(var)3*–*7* mutations strongly impair repression of *P*-transposable element induced gonadal dysgenesis but have minor effects on other TEs. Su*(var)3*–*7* mutations reduce piRNA cluster transcription and slightly impact ovarian piRNA production. However, this modest piRNA reduction does not correlate with transposon de-silencing, suggesting that the moderate effect of *Su(var)3*–*7* on some TE repression is not linked to piRNA production. Strikingly, *Su(var)3*–*7* genetically interacts with the *piwi* and *aubergine* genes, key components of the piRNA pathway, by strongly impacting female fertility without impairing transposon silencing. These results lead us to propose that the interaction between *Su(var)3*–*7* and *piwi* or *aubergine* controls important developmental processes independently of transposon silencing.

## Introduction

Constitutive heterochromatin is a nearly universal component of eukaryotic genomes. Heterochromatic regions are late replicating, more condensed, predominantly located near centromeres and telomeres, and contain only a few genes. They are associated with specific proteins as, in Drosophila, methylated H3K9, HP1, a chromo domain protein, Su(var)3–9, a histone-methyltransferase responsible for H3K9 methylation and Su(var)3–7. Su(var)3–7 is a seven zinc-finger domains protein that has affinity for DNA [1], [2], localizes mainly at centromeric heterochromatin [3], [4], and physically and genetically interacts with HP1 and Su(var)3–9 [4]–[6].

Heterochromatin DNA is mostly composed of repetitive sequences, including transposable elements (TEs) and satellite sequences. TEs represent a conspicuous fraction of eukaryotic genomes, varying from 3% in yeast to 15% in *Drosophila*, 45% in humans, and up to 90% in some plants (reviewed in [7]). Active TEs are highly mutagenic, often targeting protein-coding genes for insertion, and causing chromosome breakage, illegitimate recombination and genome rearrangements. Transposon control is especially critical in the germline, where transposon activity can create a mutational burden transmitted to subsequent generations. Most TEs are however kept silenced by the host genome. Genomic sites containing full-length or defective copies of TEs can establish a complete repression of the other copies of the same family scattered throughout the genome [8]. This repression was shown to occur *via* small RNAs mediated silencing. In Drosophila gonads, small RNAs of 23–30 nucleotides in length, called piRNAs, are derived from transposons and repetitive elements dispersed in the genome [9]. piRNAs bind proteins of the Piwi subfamily of Argonaute proteins, and serve as guide to silence their targets through complementary base-pairing [10], [11].

In *Drosophila*, the primary sources of piRNAs are discrete genomic regions called piRNA clusters, mainly localized into heterochromatin [12]. These loci are composed of repetitive sequences, transposons and inactive transposon remnants. piRNA clusters are transcribed in long precursor transcripts which are processed in the cytoplasm into small piRNAs. In the nurse cells, the piRNAs processing occurs in a diffuse structure surrounding the nucleus called the nuage. In the somatic follicle cells, it occurs in structures called Yb bodies. It was first proposed that transposon repression was essentially post-transcriptional, as a result of transcript degradation. But it was later shown that mature piRNAs loaded onto Piwi can enter the nucleus and silence their targets *via* transcriptional gene silencing.

Do heterochromatin factors play a role in transposon silencing? The role of heterochromatin was seen mostly at the level of piRNA cluster expression. SetDB1, which places the heterochromatic H3K9methylated mark in ovaries, and *rhino*, a germline specific HP1 homolog, are required for expression of double-strand RNA producing clusters [13], [14]. ChIP analysis showed HP1 binding to several piRNA clusters [15] and its requirement for production of telomeric cluster piRNAs [16]. Recently however, new evidence demonstrated that Piwi silences transposons also by repressing their transcription: piRNAs guide Piwi to its target loci, where it recruits enzymes that establish silencing [17]–[20]. Specifically, depletion of Piwi increases the amounts of RNApolII on transposon sequences and reduces the level of H3K9me3 and HP1 [17], [19], [21], [22]. Heterochromatin is thought to play not only an essential role in the transcriptional silencing of transposons, but also on the expression of nearby genes due to spreading of heterochromatic marks from transposon insertion into flanking genomic sequences [17], [19]. Recent genetic screens aimed at identifying factors of the piRNA mediated silencing pathway have confirmed the implication of Su(var)3–9 and HP1, and have revealed new chromatin factors as His2av, Lsd1 and Su(var)2–10 [23]–[25]. However, the nature and precise role of these factors involved in the silencing effector step are still unknown. The impact of *HP1* mutation on transposon silencing is modest [20], and H3K9me3 itself does not seem to be the final silencing mark [19]. Interestingly, proteomic analysis of Piwi complexes isolated from *Drosophila* ovaries did not identify heterochromatin-associated factors [21]. Recently, the three Piwi proteins, Piwi, Aubergine and Ago3, were shown to be essential for early *Drosophila* embryogenesis [26]. Embryos maternally depleted for any one of the three proteins display severe mitotic defects, and chromatin disorganization is furthermore observed in absence of maternal Piwi protein. This supports our confidence in an essential somatic function oustside the germline for the piRNA pathway.

We have examined here the role in ovaries of Su(var)3–7, an essential component of heterochromatin in *Drosophila melanogaster*, and a companion of HP1. We present evidences that *Su(var)3*–*7* is involved in oogenesis, female fertility and in the first embryonic divisions. In the ovaries, Su(var)3–7 accumulates in heterochromatic domains of somatic and germline nuclei, where it co-localizes with HP1. Absence of Su(var)3–7 induces *P*-element female dysgenic sterility but only weakly affects silencing of other TEs. We show that *Su(var)3*–*7* regulates piRNA cluster expression and modestly impacts the piRNA production. This modest piRNA reduction does not however correlate with transposon de-silencing. Strikingly, we also report that *Su(var)3*–*7* genetically interacts with *piwi* and *aubergine*, by strongly affecting female fertility without impairing TE silencing. This suggests that, independently of TE regulation, *Su(var)3*–*7* impacts the piRNA pathway to control important biological processes related to genome stability.

## Materials and Methods

### Fly Stocks

#### Su(var)3–7 mutants

*Su(var)3*–*7^R2a8^*, *Su(var)3*–*7^14^* and *Su(var)3*–*7^9^* were obtained in two different homologous recombination experiments [6], [27]. *Su(var)3*–*7^R2a8^* and *Su(var)3*–*7^14^* behave genetically as null mutations, their phenotypes at the homozygous state being identical to those of the hemizygous state over the gene deficiency [6], [27]. In contrast, the *Su(var)3*–*7^9^* allele is genetically hypomorphic (6). The *NA-P*(1A) line is described in [28]. The *piwi^1^* and *piwi^2^* lines were kindly provided by H. Lin. The *aub^QC42^*/*CyO* line was obtained from the Bloomington Stock Center [29].

### Fecundity/Fertility test of Su(var)3–7 mutant females

40 *w^1118^* and *Su(var)3*–*7^R2a8^* females were individually crossed at 25°C with wild type males. With the viable *Su(var)3*–*7^9^* allele, 40 females were crossed with males of the same genotype. In each case, the total number of eggs laid over a period of 8 days and the total number of pupae and adults that developed from these eggs were counted.

### Ovary and embryo staining

*Drosophila* embryos were collected on agar plates, dechorionated 2 minutes in bleach and fixed as previously described [30]. Ovaries were dissected from 3–4-days old females in PBS. Ovaries were then fixed as described in [31]. Briefly, ovaries were fixed one minute in 1.4% paraformaldehyde and 50% heptane, and another 20 minutes in 3% paraformaldehyde and 5% DMSO. Ovary were next rinse twice in 100% methanol, washed 1 hour in PBT 0.5% Triton-X100 and in PBT 0.5% Tween20, and another hour in PBT 0.3% Triton-X100 and in PBT 0.3% Tween20. Immunostainings were done overnight at 4°C in blocking solution with primary antibodies used at the following dilutions: rabbit anti-Su(var)3–7 [3], 1∶100; mouse anti-HP1 (C1A9, DSHB), 1∶100; mouse anti-Piwi (P4D2, kindly provided by K. Saito), 1∶2; mouse anti-Histone (MAB052, Millipore), 1∶500; rabbit anti-H3S10p (06-570, Millipore), 1∶500; rabbit anti-H3K9me3 (07-442, Millipore), 1∶100; rabbit anti-Vasa (kindly provided by R. Lehmann), 1∶4000; rabbit anti-H3K14ac (06-911, Millipore), 1∶300; mouse anti-HA (16B12, Covance), 1∶200. Samples were next washed in PBT and incubated with 1∶400 dilutions of the following secondary antibodies: Alexa Fluor-555 conjugated goat anti-mouse (A21422, Invitrogen) and Alexa Fluor-488 conjugated goat anti-rabbit (A11008, Invitrogen). Finally, samples were washed in PBT and DNA was counterstained with 0.1 µg/ml DAPI. Samples were mounted in Vectashield, and imaged either by confocal microscopy (Leica TCS SP2 AOBS) or by wide-field microscopy (Zeiss Axioplan).

### Quantitative RT-PCR analysis

RNAs were extracted from at least 30 ovary pairs of 3–4-days old flies with Trizol followed by DNase treatment, and quality of the RNA samples was assessed by Agilent 2100 Bioanalyser (Agilent Technologies Inc, Palo Alto CA). cDNA were prepared using random priming of 0.5 to 1 µg of total RNA and the PrimeScript (TAKARA BIO Inc.) or the SuperScript III RT (Invitrogen) enzymes. Quantitative PCRs were performed using Power SYBR Green Master Mix (Applied Biosystems), on a SDS 7900 HT instrument (Applied Biosystems). Each experiment was performed in biological triplicates and technical triplicates. Relative RNA levels were calculated using the GeNorm method [32] and normalized to the four control genes *tubulin α*, *rp49*, *Ef1g* and *Gapdh1*. All real-time PCRs were performed at the Genomics Platform, NCCR “Frontiers in Genetics” (http://www.frontiers-in-genetics.org/genomics.html). Primers used for qPCR are listed in Table S2.

### Immunostaining of pseudonurse cells polytene chromosomes

Polytene chromosomes from ovarian nurse cells of *otu^11^* mutants were dissected and squashed according to [33]. Slides were hybridized with a rabbit anti-Su(var)3–7 [3] at 1∶10 dilution, and DNA was counterstained with DAPI.

### Synthesis of *NA-P*(1A); *Su(var)3*–*7*/*Sb* lines

The *NA-P*(1A) line and the *w^1118^*; *Su(var)3*–*7^R2a8^*/*Sb* and *yw* ^c^; *Su(var)3*–*7^R14^*/*Sb* balancer lines were used to generate new established lines carrying the *NA-P*(1A) telomeric element at the homozygous stage and a *Su(var)3*–*7* mutant allele maintained over a floating balancer third chromosome marked with *Sb*. Autosomal substitution was performed using the *M5*; *Cy*/*T(2;3)ap^Xa^* and *yw*; *Cy*; *TM3, Sb*/*T(2;3)ap^Xa^* M lines. At each generation, the *NA-P*(1A) telomeric *P* element was maternally inherited in order to maintain strong *P* repression capacities. All crosses were performed at 25°C. In order to have accurate controls for *NA-P*(1A) repression capacities in a *Su(var)3*–*7^+^* background with the presence of a *Sb* balancer chromosome, *NA-P*(1A); +/*TM3, Sb* lines were simultaneously synthetized. They were maintained further by selecting Sb phenotypes at each generation. Once established, the three types of lines (*NA-P*(1A); +/*Sb* line, *NA-P*(1A); *Su(var)3*–*7^R2a8^*/*Sb* line and *NA-P*(1A); *Su(var)3*–*7^R14^*/*Sb* line) were maintained at least five generations before the experiment to be performed to allow an equilibrium to be reached for *P* repression capacities. Before starting the experiment, a “G_0_” cross was also performed for each line with only [Sb] individuals in order to homogenize the conditions with regard to *Su(var)3*–*7*.

### Gonadal dysgenesis assay

The ability of females to repress the occurrence of gonadal dysgenic sterility (GD sterility) was measured by the “A* assay” [34]. Tested females were crossed with strong P males (Harwich-2). For each test cross, 3 pairs were mated and immediately placed at 29°C. Parents were discarded after three days of egg-laying. Approximately two days after hatching, progeny was collected and allowed to mature for two days. 25 to 50 female progenies were then taken at random for dissection. Dissected ovaries were scored as unilaterally atrophic (S1 type) or bilaterally atrophic (S0 type) [35]. The frequency of gonadal dysgenesis was calculated as %GD = %S0+1/2%S1 and will be referred to as percentage of GD A* (%GD A*). The M cytotype, which allows *P* elements to be active, results in a high percentage of GD A*, whereas the P cytotype, which represses *P* element activity, results in a low percentage of GD A* (<5%). An intermediate percentage indicates incomplete repression. In each set of experiments, as a control for the Harwich-2 reference P strain and the experimental conditions, M females (Canton^y^) were crossed at 29°C with Harwich-2 males and 50 G_1_ females were scored for GD sterility: in each case, 100% GD sterility was observed. In addition, lines carrying *Su(var)3*–*7* mutant alleles, which are devoid of natural *P* elements (M genetic background), were as expected also found to be completely devoid of repression capacities, as tested by GD A* assay. GD sterility percentages produced in different genetic contexts were compared using the non-parametric Mann-Whitney test performed on A* assay replicates.

### Small RNA sequencing and analysis

Small RNAs were extracted from heterozygous and homozygous *Su(var)3*–*7^R2a8^* mutants ovaries using RNeasy Mini Kit (Qiagen) followed by DNase treatment. RNA fractions ranging from 15–30 nucleotides were isolated and purified from 15% polyacrylamide gels, modified by sequential ligation of 3′ and 5′ adapters. The constructs were purified again on an acrylamide gel to remove emtpy adapters and then reverse-transcribed and PCR-amplified. High-throughput sequencing was performed on a Illumina HiSeq 2000 (1×50 cycles) (FASTERIS SA, Switzerland). After demultiplexing and adapter removal, 8.79 M and 15.89 M reads were obtained for these libraries. The libraries are deposited at GEO (Gene Expression Omnibus) under the accession number GSE53015 (http://www.ncbi.nlm.nih.gov/geo/query/acc.cgi?acc=GSE53015). The resulting libraries were analyzed as previously described [12]. Only piRNA populations ranging from 23–29 nt matching the *Drosophila melanogaster* genome release 5.47 were considered for downstream analysis. Inserts mapping to piRNA clusters (perfect matches, unique position) and transposon sequences (allowing 1 mismatch) were identified using Samtools according to [12]. Quantification of inserts overlapping the piRNA clusters and transposon sequences was done using SeqMonk V0.19 software (Babraham Bioinformatics), and libraries were normalized for comparison to miRNAs (see Figure S4). Post-processing of the raw counts was performed using R 2.7.1 software (R Foundation for Statistical Computing).

## Results

### Su(var)3–7 accumulates in somatic and germline cells of ovaries and localizes mainly to heterochromatin

**Figure 1:**
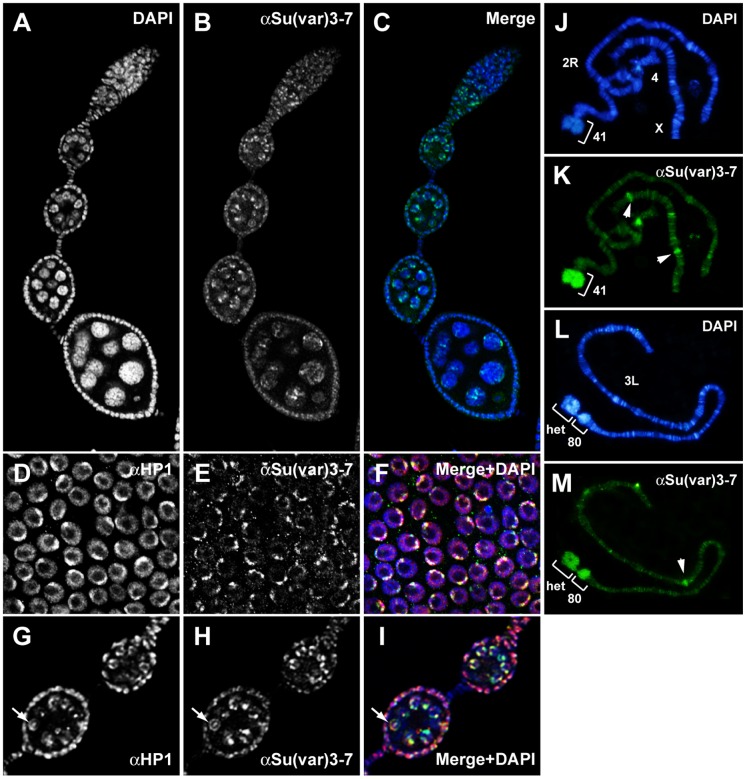
*Su(var)3*–*7* is expressed in somatic and germline cells of wild type ovaries and colocalizes with HP1. (**A**–**C**) Confocal images of wild type ovary stained with anti-Su(var)3–7 (green) and DAPI (blue) for DNA visualisation. Same procedure on *Su(var)3*–*7^R2a8^* homozygous ovaries shows complete absence of Su(var)3–7 staining (not shown). (**D**–**F**) Focus on somatic follicular cells and (**G**–**I**) germline cells co-stained with anti Su(var)3–7 (green) and anti-HP1 (red) antibodies. Arrows indicate the germinal vesicle with the karyosome. (**J**–**M**) Polytene chromosomes from *otu^11^* pseudonurse cells labelled with anti-Su(var)3–7 (green) and DAPI (blue). *otu* mutation causes polytenization of nurse cells chromosomes allowing mapping of chromosome-associated proteins [59]. Su(var)3–7 binds centromeric heterochromatin (bracket) and several euchromatic sites scattered along the chromosome arms (arrowheads).

We investigated Su(var)3–7 accumulation and localization in ovaries by immunodetection with an anti-Su(var)3–7 antibody [3]. Su(var)3–7 is detected in the nucleus of both somatic and germline cells, from the germarium until late stages of oogenesis (Figure 1A–C). Specifically, Su(var)3–7 accumulates in DAPI bright regions of the nucleus of follicular cells (Figure 1E,F). In germ cells, Su(var)3–7 is distributed in several foci within the nucleus of nurse cells, and accumulates in the germinal vesicle and on the karyosome of the oocyte (Figure 1H,I). Double immunostaining with antibodies against HP1 showed that Su(var)3–7 is localized within HP1 enriched regions in most of somatic (Figure 1D–F) and germline (Figure 1G–I) nuclei. We next determined the binding pattern of Su(var)3–7 on pseudonurse cell polytene chromosomes from *otu* mutant. Nurse cells of females homozygous for *otu* alleles contain giant polytene chromosomes, providing a unique system for studying chromosomal binding of proteins during oogenesis Mapping results are summarized in Table S1 and are illustrated in Figure 1 (J–M). On pseudonurse cell chromosomes, Su(var)3–7 is detected in all pericentromeric regions. In addition, the protein binds at least 92 sites along the euchromatic arms (about 20 sites by arms, Table S1), more than those found in salivary gland chromosomes [3]. Among Su(var)3–7 euchromatic sites, 73% are also bound by HP1 and 58% are shared by the three partners of constitutive heterochromatin, Su(var)3–9, HP1 and Su(var)3–7 (Table S1). These data indicate that Su(var)3–7 is a component of heterochromatin in germline and somatic cells.

### *Su(var)3*–*7* mutations impair female fecundity, fertility, egg chambers integrity and embryonic development

*Su(var)3*–*7* null mutations cause a maternal recessive lethality [6], [27]. When homozygous *Su(var)3*–*7* null mutant females and males are crossed together, all the progeny dies during the first or second instar larval stage. When however crossed to wild type males, homozygous mutant females are still fertile but produce limited amounts of progeny [27]. We investigated in more details the impact of the lack of maternal *Su(var)3*–*7* activity on female fertility.

**Figure 2:**
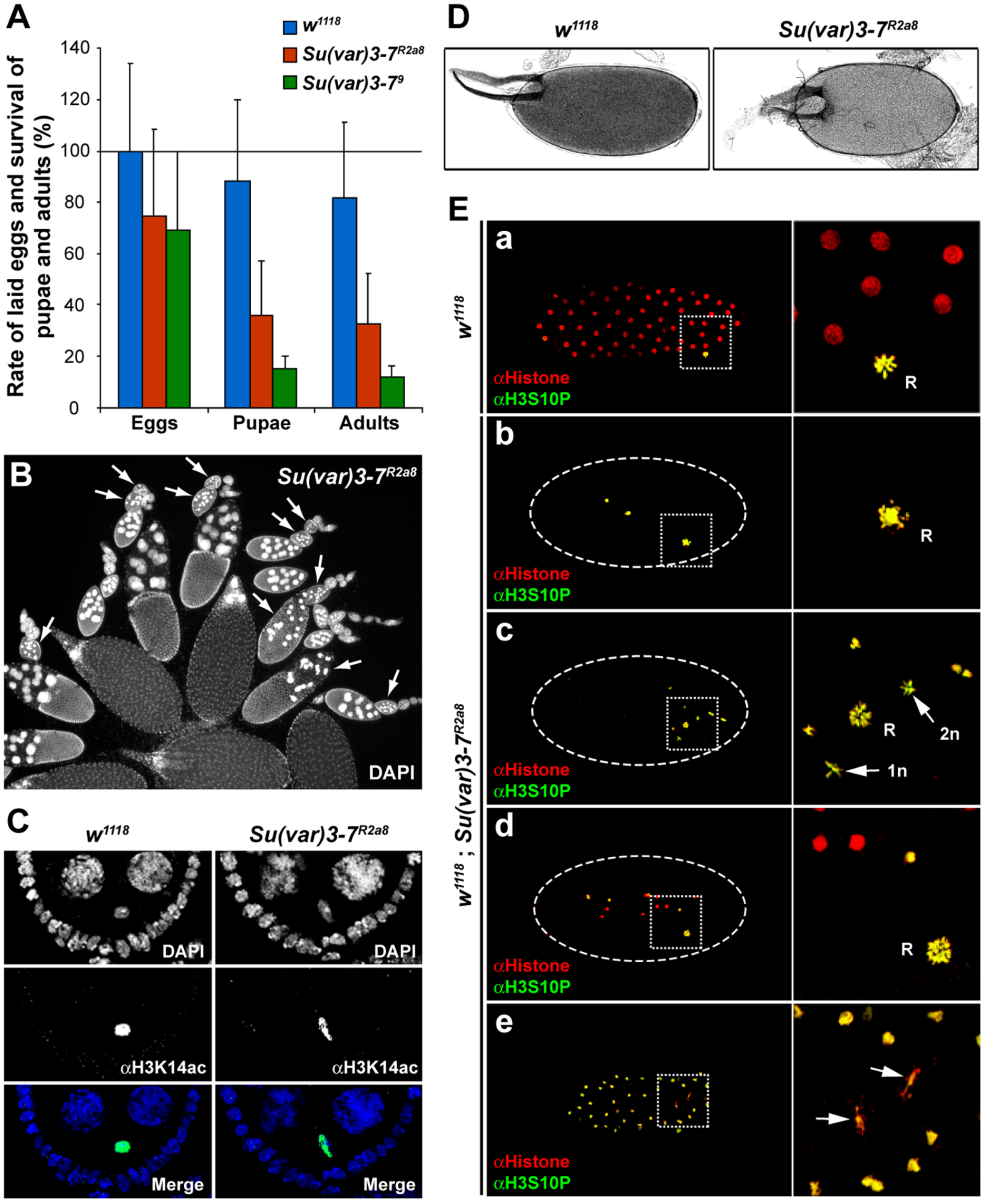
*Su(var)3*–*7* is required for oogenesis, embryogenesis and female fertility. (**A**) Fertility test of control (*w^1118^*) and *Su(var)3*–*7^R2a8^* and *Su(var)3*–*7^9^* homozygous mutant females. Bars represent the rate of laid eggs per female and the viability at pupal and adult stages. Error bars indicate the standard deviation, n = 40. (**B**) DAPI staining of a 3 days old *Su(var)3*–*7^R2a8^* mutant ovary. Arrows indicate degenerated egg chambers. (**C**) Confocal pictures of stage 5 egg chambers labelled with anti-H3K14ac antibody (green), DNA was visualized by DAPI (blue) staining. The round-shaped oocyte nucleus (karyosome) observed in the control (*w^1118^*, left panel) is altered in *Su(var)3*–*7* mutant ovary (right panel). (**D**) Phase-contrast images of mature eggs produced by control (*w^1118^*) and *Su(var)3*–*7^R2a8^* mutant females. (**E**) *Su(var)3*–*7* loss-of-function causes meiosis defects and embryonic development arrest. (**a**–**e**) Confocal images of (**a**) control (*w^1118^*) and (**b**–**e**) *Su(var)3*–*7^R2a8^* mutant embryos stained with an anti-H3S10P (green) used as mitotic marker and anti-core histone proteins (red) antibodies. Embryos were examined 60′ to 120′ AED to ensure that control embryos have reach and exceeded mitotic cycle 6. (**a**) Mitotic cycle 7/8 control embryo. The nuclei are uniformly distributed within the embryo and the three female polar bodies are assembled into a single rosette. (**b**) Mutant embryo arrested at mitotic cycle 1. The rosette is misassembled and fragmented. (**c**) Mutant embryo arrested at mitotic cycle 3. The nuclei remain localized in the anterior part of the embryo and some nuclei contain a single set of chromosomes (1 n) suggesting cases of haploid mitotic cycles. (**d**) Mutant embryo arrested at mitotic cycle 4. The nuclei divided asynchronously. (**e**) Mitotic cycle 6 mutant embryo. Arrowheads point damaged mitotic nuclei. R, rosette.

To determine female fecundity and fertility, homozygous *Su(var)3*–*7* mutant females were crossed individually with wild type males. The total number of eggs laid over a period of eight days and the total number of pupae and adults that developed from these eggs were counted. The *w^1118^* line from which the *Su(var)3*–*7^R2a8^* null allele was obtained by homologous recombination [27] was used as control (Figure 2A). Homozygous *Su(var)3*–*7^R2a8^* mutant females lay almost 30% less eggs than control females (n = 40), a significant decrease of fecundity. From these eggs, 52% (n = 3170) do not reach the pupal stage, whereas 88% of wild type eggs (n = 4261) develop into pupae (Figure 2A). Homozygous *Su(var)3*–*7^R2a8^* females give rise to only 40% adult flies compared to control females, even when a wild type dose of the gene is brought in by the father. This shows that the lack of maternally provided Su(var)3–7 protein is only partially rescued by zygotic *Su(var)3*–*7* expression. The hypomorphic *Su(var)3*–*7^9^* allele [27], although homozygous viable, also displays reduced female fertility since an homozygous *Su(var)3*–*7^9^* female crossed with *Su(var)3*–*7^9^* males produces in average only 14% (n = 466) of the expected adult progeny (Figure 2A). We concluded that the absence or reduced amounts of Su(var)3–7 impairs female fecundity and development of the embryos.

To examine the cause of the reduced female fertility, we analyzed the morphology of ovaries. Females homozygous for the null *Su(var)3*–*7^R2a8^* or hypomorphic *Su(var)3*–*7^9^* mutations display ovaries with frequent (14%, n = 2382) degenerating egg chambers during oogenesis (Figure 2B and Table 1). In degenerated egg chambers, nuclei are smaller, more DAPI-dense, and chromatin has an abnormal appearance, being more compact and fragmented. Interestingly, this phenotype is germline specific, as somatic follicular cells around the degenerated egg chamber appear normal. Degeneration occurs at various stage during egg chambers maturation, and more than 60% of the ovarioles contain at least one degenerated chamber. In *Su(var)3*–*7* mutants, karyosome formation is affected in 13% of the egg chambers (n = 1502). Instead of forming a compact spherical structure, the oocyte DNA assumes various shapes, often elongated or separated in clumps (Figure 2C). Degenerating egg chambers and karyosome defects are rescued by the expression of a wild type tagged version of the protein expressed under the control of its own promoter, the *P[HA-Su(var)3*–*7]* construct (Table 1; Figure S1 and supporting Materials and Methods in File S1). This shows that these ovary phenotypes are due to the lack of Su(var)3–7 and not to a second site effect. Finally, 13% (n = 473) of the mature eggs produced by *Su(var)3*–*7^R2a8^* or *Su(var)3*–*7^9^* female are abnormal. The eggs are small and round with shortened dorsal appendages (Figure 2D), and a few of them (2%) are ventralized with closed or even fused dorsal appendages. Although 85% of the embryos laid by *Su(var)3*–*7* mutant females appear wild-type, 52% of them will not reach adult stage. We wondered whether the lack of Su(var)3–7 impairs heterochromatin in ovaries. Immunodetection of HP1 and H3K9me3 in ovaries lacking Su(var)3–7 does not reveal modifications of the pattern and the amounts of these two heterochromatic markers (Figure S2). We conclude that absence of Su(var)3–7 does not visibly impair heterochromatin in adult ovaries.

**Table 1:** Rescue of *Su(var)3*–*7^R2a8^* ovarian phenotypes by expression of *P[HA:Su(var)3*–*7]*.

Genotype	Degenerating egg chambers (%)	Defective karyosomes (%)
*w^1118^*	2.3 (n = 1148)	1.5 (n = 854)
*w^1118^*; *Su(var)3*–*7^R2a8^*/*TM6*	5.1 (n = 1136)	1.7 (n = 231)
*w^1118^*; *Su(var)3*–*7^R2a8^*	14.2 (n = 2382)	12.7 (n = 1502)
*w^1118^*; *Su(var)3*–*7^R2a8^*; *P[HA:Su(var)3*–*7]*	5.8 (n = 1292)	2.1 (n = 811)

n indicates the number of examined egg chambers.

To address the cause of embryonic development arrest, we examined early embryos laid by homozygous *Su(var)3*–*7^R2a8^* females. 25% (n = 1520) of the one to two hours old embryos are blocked at the preblastoderm stage, versus 4% for wild type. We observed distinct phenotypes among them 15% contain a misassembled and fragmented rosette, meaning that integrity of the meiosis products (the three polar bodies) is impaired (Figure 2Eb). Some others contain an apparently normal rosette but nuclei are not uniformly distributed within the embryo or clearly contain different amounts of DNA, reflecting loss of material or haploid mitotic cycle (Figure 2Ec). In other cases, chromosome bridges and lagging chromosomes with dispersed chromatin fragments were observed in anaphase and early telophase figures (Figure 2Ee). Finally, quite frequently, nuclear divisions appear asynchronized (Figure 2Ed). Altogether these phenotypes show that the lack of maternal Su(var)3–7 leads to defects in meiosis and first mitotic divisions, due to chromatin fragmentation or chromosome loss, implicating Su(var)3–7 as an important actor for maintenance of chromosome integrity.

### Su(var)3–7 is required for the silencing of the *P* transposable element, but has a minor effect on other TEs

We showed above that Su(var)3–7 is associated with heterochromatic regions of ovarian cells nuclei where it colocalizes with HP1. As heterochromatin is mainly composed of transposons and since HP1 is implicated in their silencing [17], [19], [20], we investigated the effect of *Su(var)3*–*7* mutations on the expression of several transposons in ovaries. We tested first its functional implication in the silencing of a well-studied *Drosophila* transposon, the *P* element. In absence of repression, high *P* element activity is responsible for a syndrome of germline abnormalities, called gonadal dysgenesis, characterized by a high mutation rate, chromosomal rearrangements, male recombination and agametic sterility, referred to as GD sterility (Gonadal Dysgenesis) [34]. *P* copies that are responsible for *P* regulation have been identified: *P* elements inserted in the heterochromatic Telomeric Associated Sequences (TAS) on the *X* chromosome (for example the *NA-P*(1A) element) strongly repress dysgenic sterility and *P* transposition [8], [28], [36]. In addition, the regulatory properties of this element can be inhibited by the deletion of the *Su(var)205* gene encoding HP1 [37]. It was thus interesting to test if the *P* repression elicited by *P*(1A) copy is sensitive to *Su(var)3*–*7* mutations.

The defective telomeric *NA-P*(1A) insertion was combined with the *Su(var)3*–*7* mutant alleles. Homozygous lines carrying the *NA-P*(1A) telomeric copy were combined with various genetic contexts including the *Su(var)3*–*7^R2a8^* and *Su(var)3*–*7^14^* homozygous and heterozygous mutants and the *Su(var)3*–*7^R2a8^*/*Su(var)3*–*7^14^* heteroallelic combination generated by the two reciprocal parental crosses. These mutant contexts are compared to the repression capacities of *NA-P*(1A) in a *Su(var)3*–*7* wild type background, with or without balancer third chromosome carrying the *Sb* marker. *P* repression capacities were tested by measuring the ability to repress the occurrence of dysgenic sterility caused by *P* elements activity (GD repression assay). Table 2 shows that *NA-P*(1A) insertion in such a *Su(var)3*–*7* wild type background has strong *P* element repression capacity, since only 1–2% of GD sterility is observed with [Sb^+^] females (a level similar to that obtained when testing the initial *NA-P*(1A) line) and we found only a weak effect of the *TM3, Sb* chromosome (around 10% of GD). With the *Su(var)3*–*7^R2a8^* allele, no dose effect was found (GD = 8.4% for the [Sb] tested females) whereas a strong effect was observed in the homozygous *Su(var)3*–*7* mutant state (GD = 82.2% for the [Sb^+^] tested females, Table 2). With the *Su(var)3*–*7^14^* allele, a nearly complete loss of repression was found in the homozygous state (GD = 97.9%), whereas a tendency to reduction was observed at the heterozygous state (GD = 47.6%). In the heteroallelic *Su(var)3*–*7* mutant contexts, again a nearly complete loss of repression occured (GD = 98.3% and 98.9%), whereas a weak tendency was observed for [Sb] females which are heterozygous for one of the two mutant alleles (GD = 23.5% and 29.8%). These results show that the loss of *Su(var)3*–*7* function severely impairs the repression capacity of the *NA-P*(1A) element and is crucial to repress GD sterility linked to *P* transposition. We provide thus strong evidence for a role of Su(var)3–7 in *P* element silencing.

**Table 2:** Effect of *Su(var)3*–*7* mutations on *P*-element repression elicited by *P* copies inserted in subtelomeric heterochromatin.

Genotype of parents	Phenotype of the female progeny tested for *P* repression capacities (%)	
	*Sb* progeny	*Sb* ^+^ progeny
F *NA-P(1A)*; *Su(var)3*–*7^R2a8^*/*Sb* M *NA-P(1A)*; *Su(var)3*–*7^R2a8^*/*Sb*	8.44 (5.3) n = 9	82.2 (13.1) n = 5
F *NA-P(1A)*; *Su(var)3*–*7^14^*/*Sb* M *NA-P(1A)*; *Su(var)3*–*7^14^*/*Sb*	47.6 (16.7) n = 8	97.9 (4.2) n = 4
F *NA-P(1A)*; *Su(var)3*–*7^R2a8^*/*Sb* M *NA-P(1A)*; *Su(var)3*–*7^14^*/*Sb*	23.5 (13.7) n = 11	98.3 (2.7) n = 10
F *NA-P(1A)*; *Su(var)3*–*7^14^*/*Sb* M *NA-P(1A)*; *Su(var)3*–*7^R2a8^*/*Sb*	29.8 (17.9) n = 13	98.9 (1.6) n = 5
F *NA-P(1A)*; *Su(var)3*–*7^+^*/*Sb* M *NA-P(1A)*; *Su(var)3*–*7^+^*/*Sb*	11.6 (7.4) n = 7	1.2 (1.5) n = 7

The *P* repression capacities of the female progeny having inherited or not a *Sb* chromosome are shown. The mean GD percentage calculated on the basis of all replicates is given with the standard deviation among replicates (in parenthesis). “n” indicates the number of replicates performed. F: female; M: male.

**Figure 3:**
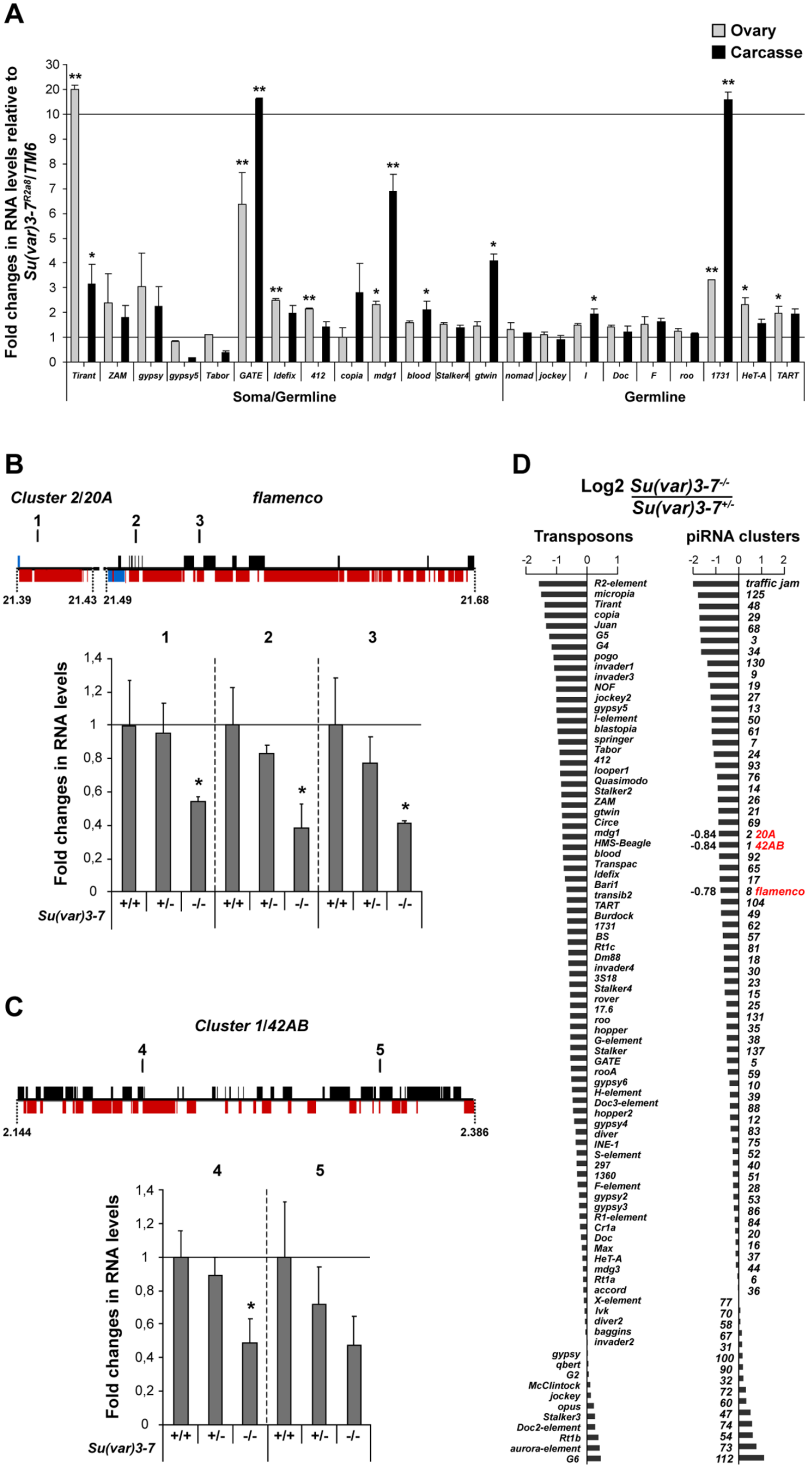
*Su(var)3*–*7* has a weak impact on transposon silencing, but regulates piRNA clusters transcription. (**A**) Quantitative RT-PCR analysis on 22 retrotransposons in *Su(var)3*–*7^R2a8^* homozygous mutant ovaries (grey bars) and female carcasses (black bars). Histograms represent the fold changes in RNA levels relative to *Su(var)3*–*7^R2a8^*/*TM6* siblings; error bars indicate the standard deviation of triplicate samples (n = 3). Differences in the fold changes were tested by a Welch t-test (* : p<0,05; ** : p<0,01). (**B**) Quantitative RT-PCR analysis of *cluster 2* and *flamenco* from control (*w^1118^*) and *Su(var)3*–*7^R2a8^* heterozygote and homozygote mutant ovaries. Shown are the fold changes in RNA levels relative to the control (n = 3; * : p<0,05). The position of the primer sets used for qRT-PCR are indicated by bars named 1, 2 and 3 along the map above. Coordinates of the clusters along the *X* chromosome are indicated in Mb. Boxes indicate protein coding genes (blue) and transposon fragments in sense (black) and antisense (red) orientation. (**C**) Quantitative RT-PCR analysis of *cluster1* from control (*w^1118^*) and *Su(var)3*–*7^R2a8^* heterozygote and homozygote mutants. Shown are the fold changes in RNA levels relative to the control (n = 3; * : p<0,05). A map of the *cluster1*/*42AB* locus with position of the qPCR primer sets 4 and 5 is shown above. (**D**) Histograms show the log2 fold ratios of normalized ovarian piRNAs mapping antisense to transposons (left) and uniquely mapping piRNAs (sense plus antisense) over piRNA clusters (right) between homozygous and heterozygous *Su(var)3*–*7* mutants. Up to 1 mismatch was allowed between reads and transposon sequences.

Encouraged by the strong effect of Su(var)3–7 on *P*, we decided to test whether others *Drosophila* transposons are kept silenced by the heterochromatic protein. TE transcription levels were measured by quantitative RT-PCR in *Su(var)3*–*7^R2a8^* homozygous mutant ovaries or female carcasses, and compared to levels monitored in *Su(var)3*–*7^R2a8^* heterozygous siblings. We tested 22 transposons belonging to germline or soma-active element [10], [11]. Surprisingly, in *Su(var)3*–*7* mutant ovaries, TEs are only moderately de-silenced. Only *GATE*, and *Tirant* are strongly derepressed in ovaries (Figure 3A). For a few other transposons, *mdg1*, *Idefix*, *412*, the increase of expression is low (a factor of two to four) and for all others the total absence of Su(var)3–7 has no impact on their expression (Figure 3A). Derepression is a bit stronger in carcasses, except for *Tirant*. These results show that Su(var)3–7 is moderately implicated in the silencing of transposons in fly. To verify this result, we have tested another *Su(var)3*–*7* mutant genetic background using the hetero-allelic combination *Su(var)3*–*7^R2a8^*/*Su(var)3*–*7^14^*. The *Su(var)3*–*7^14^* allele, obtained in a previous homologous recombination screen, is also considered as a null mutation [27]. Very similar results were observed (Figure S3A) removing the possibility of a second site effect on TE expression in the homozygous *Su(var)3*–*7^R2a8^* mutants. We conclude that the absence of functional Su(var)3–7 in ovaries and carcasses leads to only a modest de-silencing of few retrotransposons, and that Su(var)3–7 is not a general factor of TE silencing.

We next wondered whether the lack of maternal Su(var)3–7 impacts more strongly transposon silencing. We compared the level of transposon expression in ovaries of *Su(var)3*–*7^R2a8^*/*TM6B* females issued from either homozygous or heterozygous *Su(var)3*–*7^R2a8^* mothers. Analysis of expression of 22 transposons in both kinds of *Su(var)3*–*7^R2a8^*/*TM6B* females did not reveal any significant variation linked to the lack of Su(var)3–7 maternal transmission (Figure S3B). Therefore, the absence of Su(var)3–7 in a female slightly affects transposon silencing in its own ovaries but does not impact transposon silencing in ovaries of surviving adult progeny having paternally inherited a wild type Su(var)3–7 allele.

### Su(var)3–7 regulates transcription levels of piRNA clusters but only slightly impacts piRNA production

The majority of piRNA clusters are pericentromeric [12] and the expression of some of them is sensitive to the amounts of heterochromatic proteins in their vicinity [13], [14]. We wondered whether Su(var)3–7 is also involved in the regulation of piRNA cluster expression. We carried out quantitative RT-PCR to measure transcript levels in ovaries from germline specific *42AB*/*cluster 1*, *20A*/*cluster 2* and its neighbor soma specific *flamenco* cluster. These three clusters localize to chromosomal regions occupied by Su(var)3–7 and its partners Su(var)3–9 and HP1 in nurse cells (Table S1). Interestingly, we observed a 2 to 2.5 fold decrease of *cluster 2* and *flamenco* transcription in *Su(var)3*–*7* null mutant ovaries (Figure 3B). Su(var)3–7 is therefore required for normal level of piRNA cluster transcription. To determine the levels of *42AB* cluster expression, we performed both classical and strand-specific qRT-PCR in ovaries homozygous or heterozygous for *Su(var)3*–*7* mutations (supporting Materials and Methods in File S1). We found that loss of Su(var)3–7 significantly reduces by half the *42AB* transcripts level on both strands (Figure 3C and S3C). We thus conclude that Su(var)3–7 is required for transcription of both bi- and uni-directionnally transcribed clusters, in both germline and somatic tissues of the gonad.

As the levels of piRNA cluster transcript are expected to impact the level of piRNAs, we analysed the piRNA population in *Su(var)3*–*7* mutant ovaries. We generated a small RNA library from homozygous *Su(var)3*–*7^R2a8^* ovaries and compared it to a library derived from heterozygotes, normalization being done on miRNAs (Materials and Methods). The analysis of the piRNA population (23–29 nt) revealed that 20% of the piRNAs are lost in the *Su(var)3*–*7* mutant library (Figure S4B). piRNAs that uniquely map to the 142 piRNA clusters of the genome were quantified in the two libraries [10]–[12]. Unfortunately, the piRNA cluster associated with the TAS sequences on the *X* chromosome is absent in our *Su(var)3*–*7* mutant line, as in the sequenced genome and in most laboratory strains. We were therefore unable to determine whether the strong impact of *Su(var)3*–*7* mutation on the *P*(1A) regulatory properties is due to regulation of the TAS expression and its piRNA production. Nevertheless, considering all other piRNA clusters, primary piRNA density unveiled a general 35% reduction of piRNAs content in homozygotes compared to heterozygote (Figure 3D). A dozen of clusters display a reduction of two to four fold, including *traffic jam* and two clusters from the fourth heterochromatic chromosome, clusters 29 and 3 (Figure 3D). In the three clusters previously analyzed, *42AB*/*cluster1*, *cluster 2* and *flamenco*, a reduction of 34.8%, 34% and 32% of piRNA content was observed in *Su(var)3*–*7* mutant ovaries, correlating with the reduction monitored by the qRT-PCR analysis of their transcripts. These observations show that Su(var)3–7 is required for the normal level of primary piRNAs production from clusters, confirming the requirement of Su(var)3–7 for cluster transcription.

We then compared the levels of piRNAs mapping to each of the 90 major TEs of *Drosophila melanogaster*, in homozygote and heterozygous *Su(var)3*–*7* mutant ovaries. The overall amounts of antisense piRNAs mapping to TEs decrease of 25% in the homozygous *Su(var)3*–*7* mutant ovaries. Only modest changes in piRNA accumulation were detected, even for *GATE*, the transposon the most up-regulated by the *Su(var)3*–*7* mutation (Figure 3D). In contrast, among the highest decrease of piRNA levels were piRNAs mapping to *copia* or *gypsy5* which are not up-regulated in *Su(var)3*–*7* mutant ovaries in our qRT-PCR analysis of transcript level (Figure 3A). These results mean that even if *Su(var)3*–*7* mutation slightly impacts piRNA production, this reduction does not account for the transposon de-silencing observed in *Su(var)3*–*7* mutant ovaries. Therefore, Su(var)3–7 is likely to impact transposon silencing at the transcriptional effector step. Our results also suggest that a reduction by half of antisense transposon piRNAs is not sufficient to cause a significant up-regulation of the element. Previous studies had shown that defective piRNA biogenesis triggers loss of Piwi, presumably because unloaded Piwi is unstable [19], [38]. As expected, given the modest impact of *Su(var)3*–*7* mutation on piRNA levels, we did not detect any modification of the amounts and localization of Piwi in *Su(var)3*–*7^R2a8^* ovaries (Figure S5).

### *Su(var)3*–*7* genetically interacts with *piwi* and *aubergine* without impairing TE silencing

Piwi is a key factor of the transposon-silencing pathway and physically interacts with HP1 [39], [40]. To determine whether *Su(var)3*–*7* interacts with *piwi*, we explored genetic interactions between mutant alleles of both genes. Bringing one copy of the null *piwi^2^* mutation in the homozygous *Su(var)3*–*7^R2a8^* mutant background dramatically decreases fertility (Table 3). When crossed with wild type males, a *piwi^2^*/*CyO*; *Su(var)3*–*7^R2a8^* female gives in average only a 1.5 adult progeny, and 45% (n = 40) of these females are fully sterile, giving no adult progeny, while only 10% (n = 40) of the *Su(var)3*–*7^R2a8^* females are fully sterile and produce in average 40% of the expected adult progeny. *piwi^2^*/*CyO*; *Su(var)3*–*7^R2a8^* females lay many eggs but they do not develop. Qualitatively similar results were observed with the *piwi^1^* null allele (not shown). These results reveal a strong genetic interaction between *piwi* and *Su(var)3*–*7* genes that greatly impacts female fertility. Interestingly, removing one dose of the other Argonaute family gene, *aubergine*, also decreases the fertility of *Su(var)3*–*7* mutant females. *aub^QC42^*/*CyO*; *Su(var)3*–*7^R2a8^* females produce only 14.3% of the expected adult progeny (Table 3). These results show that, as with *piwi*, *Su(var)3*–*7* genetically interacts with *aubergine* to ensure full female fertility.

**Table 3:** *Su(var)3*–*7* genetically interact with *piwi* and *aubergine* for female fertility.

Genotype	Female sterility^a^ (%) (n = 40)	Average number of progeny/female^b^	Ventralized embryos (%)
*w^1118^*	2.5	124	0 (n = 734)
*piwi^2^*/*CyO*	5	81.2	0.26 (n = 744)
*Su(var)3*–*7^R2a8^*	10	49.8	2 (n = 368)
*piwi^2^*/*CyO*; *Su(var)3*–*7^R2a8^*	45	1.5	6.6 (n = 198)
*aub^QC42^*/*CyO*	9.8	88.4	0 (n = 631)
*aub^QC42^*/*CyO*; *Su(var)3*–*7^R2a8^*	16.6	17.8	0.46 (n = 435)

a Percentage of female giving no progeny when crossed with WT male.

b Adult progeny obtained from a period of 8 days egg laying at 25°C.

To investigate the causes of female sterility, we examined *piwi^1 or 2^*/*CyO*; *Su(var)3*–*7^R2a8^* ovaries. Globally, ovaries have normal size, display all steps of egg chamber development and produce mature eggs. Degenerated egg chambers and karyosome shape defects were observed at levels similar to those in *Su(var)3*–*7* mutants (not shown). Embryo ventralization, although slightly increased in the double *piwi^2^*/*CyO*; *Su(var)3*–*7^R2a8^* mutant (6.6%, n = 198, Table 3), cannot explain the sterility of these females. The great majority of embryos laid by *piwi^2^*/*CyO*; *Su(var)3*–*7^R2a8^* females (98%; n = 480) do not reach the larval stage, highlighting the requirement of *Su(var)3*–*7* and *piwi* genetic interaction for proper *Drosophila* embryogenesis. Knowing that Piwi is required, in part, to establish heterochromatin [17], [19], [21], [22], we wondered whether heterochromatin is impaired in this double mutant context. No obvious modification of the pattern of Piwi and of the two heterochromatic markers, HP1 and H3K9me3, was seen in immunostained ovaries from *piwi^2^*/*CyO*; *Su(var)3*–*7^R2a8^* females (not shown). This indicates that Piwi expression and heterochromatin are not visibly affected in ovaries of these sterile females.

**Figure 4:**
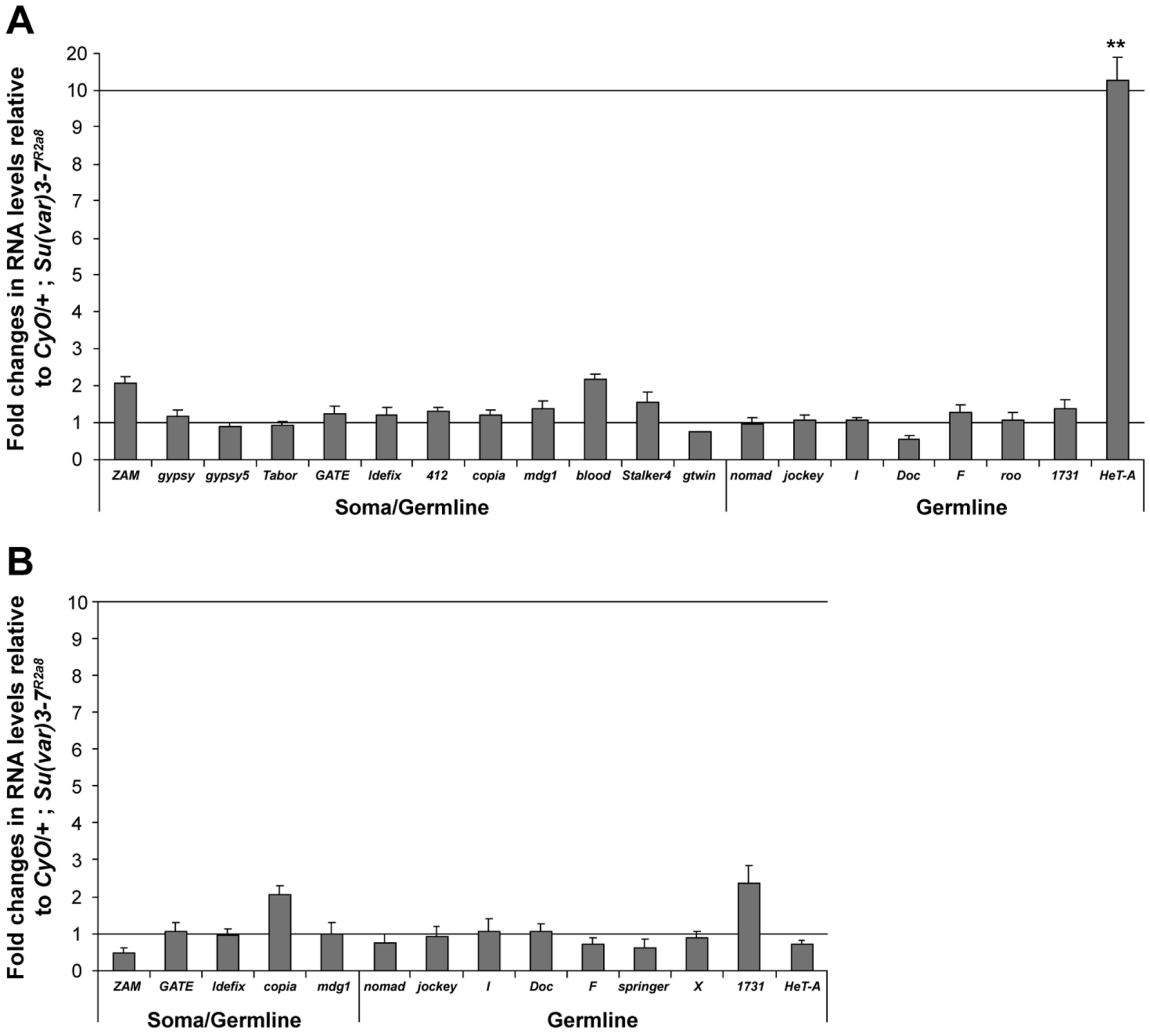
*Su(var)3*–*7* does not interact with *piwi* and *aubergine* for transposon silencing in ovaries. Quantitative RT-PCR analysis on the indicated transposons in (**A**) *piwi^2^*/+; *Su(var)3*–*7^R2a8^* and (**B**) *aub^QC42^*/+; *Su(var)3*–*7^R2a8^* ovaries. Bars represent the fold changes in RNA levels relative to *Su(var)3*–*7^R2a8^* siblings (n = 3; ** : p<0,01).

We finally tested whether female sterility is due to a strong transposon up-regulation by analyzing transposon expression in *piwi^2^*/*CyO*; *Su(var)3*–*7^R2a8^* ovaries. We asked whether removing one dose of *piwi*, which by itself does not perturbate transposon silencing (not shown), aggravates transposon de-silencing in combination with homozygous *Su(var)3*–*7* mutation. Comparison of the levels of transposon transcripts in *Su(var)3*–*7^R2a8^* and *piwi^2^*/*CyO*; *Su(var)3*–*7^R2a8^* ovaries does not reveal any difference for all the transposons examined, except for *HeT-A* (Figure 4A). This is evidence that the majority of transposons are not more de-repressed in the double mutant compared to the single mutant. The TE transcripts levels comparison was done on *aub^QC42^*/*CyO; Su(var)3-7^R2a8^* ovaries versus *Su(var)3*–*7^R2a8^* ovaries. None of the analyzed transposons were significantly more up-regulated in the double mutant than in the single mutant (Figure 4B). We conclude that *aub^QC42^*/*CyO; Su(var)3*–*7^R2a8^* reduced female fertility and *piwi^2^*/*CyO*; *Su(var)3*–*7^R2a8^* female sterility are not likely to be due to transposon up-regulation. This suggests that *Su(var)3*–*7* interacts with genes of the Piwi family for other crucial biological process ensuring female fertility and embryo development.

## Discussion

### Su(var)3–7 functions during oogenesis and early embryogenesis

We investigated the requirement for *Su(var)3*–*7* function in the *Drosophila* ovary. Consistent with previous observations performed in somatic tissues [3], the Su(var)3–7 protein localizes mainly into the nucleus of both follicular cells and germline cells throughout oogenesis and, as shown by staining of pseudonurse cells polytene chromosomes, binds preferentially to heterochromatic domains, where it co-localizes with HP1. Su(var)3–7 also colocalizes with HP1 at a number of euchromatic sites, but with some differences. Interestingly, the number of sites bound by Su(var)3–7 and HP1 on nurse cell chromosomes is higher than on salivary glands chromosomes [3], [41], suggesting an important role of the two heterochromatic proteins in oogenesis. ChIP experiments could define the genomic targets of the proteins and hence the significance of these euchromatic sites. Attesting of the important role of Su(var)3–7 in oogenesis and early embryogenesis, loss of *Su(var)3*–*7* function impairs both female fecundity and fertility, as a significant proportion of maturing eggs degenerate during oogenesis, and approximately 40% of the laid eggs do not develop into adults.

While exploring for phenotypes in *Su(var)3*–*7* mutant ovaries, we noted recurrent defects in the oocyte nucleus. Instead of forming a round condensed structure, mutant karyosomes are mispackaged, and display abnormal or fragmented shapes. Alterations of the meiosis products were also observed in mature eggs and early maternally Su(var)3–7 depleted embryos, as indicated by frequent breakages of the polar bodies. Additionally, defective mitosis leading to aberrant chromosome segregation, chromosome loss and asynchrony were also identified during the first cleavage divisions, preventing subsequent development of viable embryos. These phenotypes underline the critical role of heterochromatic factors in the maintenance of genome integrity during development (reviewed in [42]). In the reproductive tissues however, it became apparent that transposon activity also induces DNA damage during meiosis as well as in early embryos. Evidences for the deleterious effects of massive transposition come from studies of gonadal dysgenesis, where the activation of specific transposons such as *P*- and *I*-elements can cause sterility [34], [43]. In our gonadal dysgenesis assay, *Su(var)3*–*7* mutant females were unable to efficiently repress the occurrence of gonadal atrophy linked to *P*-element transposition. Mutations of many members of the piRNA pathway correlate with chromosome integrity phenotypes or meiosis and/or mitosis defects. Karyosome shape defects have been described for most of the piRNA pathway mutants [44]–[48], as was fragmentation of the zygotic genome during the first embryonic divisions [49]. In addition, germline *piwi* null alleles lead to maternal effect embryonic lethality and severe chromosome defects during the cleavage divisions [26], [50]. However it is not yet clear whether the DNA instability described in piRNA mutants is due to massive transposition. A growing number of studies suggest that the Piwi-piRNA pathway functions not only to repress transposons, but also to regulate chromatin architecture and protein-coding genes [51]. Piwi proteins function in regulating maternal transcript destruction during the maternal to zygotic transition in early embryos [52]. Targeted piRNAs impact nearby host gene transcription via transcriptional silencing of transposons [17], [19]. *piwi* deficiency modifies the distribution of several epigenetic marks all along the chromosomes, including HP1, making the Piwi-piRNA mechanism an epigenetic programming mechanism in *Drosophila* [17]. This raises the possibility that meiosis and mitosis phenotypes of piRNA pathway mutants are in fact due to changes in the global chromatin architecture more than to massive transpositions at this stage [26]. ChIP experiments using antibodies to epigenetic marks that control chromatin organization could test further this hypothesis. It would be of interest to analyze methylated or acetylated histones distribution during oogenesis and in the young embryos issued from piRNA pathway or *Su(var)3*–*7* mutants. These experiments could lead to advances in the knowledge of the role of these proteins in global chromatin organization. Our finding that the TEs are not more up-regulated in the *piwi*/+; *Su(var)3*–*7* and *aub*/+; *Su(var)3*–*7* mutant females than in *Su(var)3*–*7* mutant females, although they are sterile or less fertile, is an additional evidence supporting that fertility phenotypes of piRNA mutants do not solely result from transposon de-repression. Our results suggest that *piwi, aub* and *Su(var)3*–*7* may be involved in important biological process related to genome stability and support the new avenue that Piwi proteins play essential functions in the embryos.

### Weak impact of *Su(var)3*–*7* mutation on transposon silencing

We provide evidence that Su(var)3–7 is involved in the regulatory properties of a *P* transposable element inserted in a sub-telomeric piRNA producing cluster. Silencing of most of the transposons probably results from the expression of several clusters, all containing small pieces of transposon homologous sequences [12]. However, in the case of the *P* element, one copy that elicited all the repressive properties was isolated by recombination in a background devoid of other *P* elements [8]. The copy is inserted in a sub-telomeric heterochromatic region called *X-TAS*, which was found later to be a piRNA cluster [53]. Our results show that the presence of Su(var)3–7 is crucial for the silencing properties of this copy at this site. This strongly suggests that *Su(var)3*–*7* mutation affects *X-TAS* expression or function. The lack of Su(var)3–7 has an important deleterious effect on *P* element regulation, whilst impact on other transposons remains moderate. Most of transposons are de-silenced by a 1.5 to 7 factor. This is in contrast to hundred-fold changes described for piRNA component mutants, such as *rhino* (100–150 fold expression of about 20% of transposon families; [13]). However, some other mutations disrupt only moderately transposon silencing, as PAPI mutant, where most of transposons are up-regulated by a factor of 2.5 to 4 [54]. Germline *piwi* knockdown does not strongly increase transposon expression, with most changes by a factor between 1.9 and 5 [20]. Similarly, germline depletion of HP1 moderately impairs transposon silencing [20]. This is in contrast with previous demonstrations of the HP1 implication in transposon transcriptional silencing [19], [21], [22], [37]. Whether moderate changes in expression reflect de-silencing remains a difficult question. Considering the impact on transposon silencing of *Su(var)3*–*7* or *HP1* mutations [20] or of many piRNA members mutations, it appears that different transposons are regulated by different genes, and that, even a factor directly involved in piRNA biogenesis does not up-regulate all TEs [23]. This raises the possibility that the moderate impact of Su(var)3–7 on transposon expression reflects its specificity for a few elements or for a few piRNA clusters.

We have shown here that *Su(var)3*–*7* mutation reduces amounts of precursor transcript of several clusters and primary piRNA production. In the same way, Setdb1, a H3K9 methyl transferase responsible for the deposition of the heterochromatin H3K9 methylated mark in ovaries, is required for cluster expression [14], and *rhino*, a germline HP1 homolog, also plays a crucial role in cluster expression [13]. This suggests that heterochromatic proteins are required for cluster expression, as for protein-coding genes localized on the heterochromatic 4^th^ chromosome or in centromeric heterochromatin [55], [56]. In this context, piRNA clusters seem to behave as heterochromatic genes, by requiring a heterochromatic context to be expressed. However, some data indicate inverse effects of heterochromatin on cluster expression [15], [57], [58]. Fine tuning probably depends on the specific cluster environment, and Su(var)3–7 is a strong candidate in this fine tuning.

The amounts of anti-sense piRNAs mapping to TEs modestly decrease by 25% in the homozygous *Su(var)3*–*7* mutant ovaries. No correlation between transposon up-regulation and significant reduction in anti-sense piRNA accumulation was established, even for *GATE* de-silenced in *Su(var)3*–*7* mutant ovaries. Although *Su(var)3*–*7* mutation slightly impacts piRNA production, this reduction does not account for the de-silencing observed for a few transposons in *Su(var)3*–*7* mutant ovaries, suggesting that Su(var)3–7 impacts transposon silencing at the transcriptional effector step. This hypothesis could be tested by examining RNA Pol II and other transcription markers occupancy, notably on the *GATE* promoter, despite the difficult problem of working on a multicopy element. This approach was used to demonstrate transposable elements chromatin silencing by Piwi and other piRNA pathway components [18], [19]. For example, loss of *Maelstrom* increases RNA Pol II recruitment, nascent RNA output and steady state RNA levels of transposons although piRNA levels and Piwi loading are largely normal [19]. In addition, in our analysis, *copia* or *gypsy5* are not up-regulated in *Su(var)3*–*7* mutant although the amount of piRNAs mapping to these elements are significantly reduced. This suggests that a reduction of up to 60% of anti-sense piRNAs mapping to a given transposon is not sufficient to cause a significant up-regulation of the element, as detected by qRT-PCR. This raises the interesting question of which reduction of piRNAs is required to drive transposon up-regulation.

It is now necessary to investigate whether other heterochromatic proteins are involved in TE silencing, and to dissect in more details the interaction linking Su(var)3–7 or HP1 to Piwi proteins.

## Supporting Information

Figure S1 **Characterization of the** ***P*** **[** ***HA:Su(var)3–7*** **] transgene.** (**A**) Schematic representation of the *HA:Su(var)3–7* construct and of the *Su(var)3–7* endogenous locus. (**B**) Western blotting on crude extracts from S2 cells transfected with (1) empty vector and (2) plasmid encoding *HA:Su(var)3–7*. Membranes were probed with either anti-HA or anti-Su(var)3–7, anti-tubulin was used as a loading control. HA-tagged Su(var)3–7 migrates as a doublet at 170 kDa as previously observed with the endogenous protein [1]. (**C**) *P*[*HA:Su(var)3*–*7*] acts as an enhancer of variegation. Adult eyes of *w^m4^*/+ and *w^m4^*/+; *P[HA:Su(var)3*–*7]* flies. (**D**) Immunostaining on polytene chromosomes from salivary glands of *yw*; *P[HA:Su(var)3*–*7]* third instar larvae stained with anti-Su(var)3–7 (green) and anti-HA (red) antibodies, DNA was visualized by DAPI (blue) staining. Endogenous and HA-tagged Su(var)3–7 proteins have similar binding pattern and localize at the chromocenter (cc), on telomeres (arrowheads) and on several euchromatic sites scattered along the chromosome arms. (**E**) In ovaries, the *P[HA:Su(var)3*–*7]* transgene localizes in somatic and germline cells similarly to endogenous protein. (**a**–**i**) Confocal images of *P[HA:Su(var)3*–*7]* expressing ovary stained with anti-HA (red) and anti-Vasa (**c**, green) or anti-H3K14ac (**f**, green) antibodies, DNA was visualized by DAPI (blue) staining. Anti-Vasa was used as germline cell marker and anti-H3K14ac to label the karyosome.
(TIF)

Figure S2 ***Su(var)3***–***7*** **mutation does not impair HP1 and H3K9me3 patterns in ovaries.** (**A**–**P**) Confocal sections crossing through the germline (**A**–**D**, **I**–**L**) and the follicular epithelium (**E**–**H**, **M**–**P**) of control (upper panel) and *Su(var)3*–*7^R2a8^* homozygous mutant (lower panel) egg chamber. Ovaries were stained with anti-HP1 (red) and anti-H3K9me3 (green), DNA was labeled with DAPI (blue). HP1 and H3K9me3 are localized mainly in heterochromatin territories of nurse cells and somatic follicular cells as well as in the karyosome (arrow); the oocyte nucleus from the *Su(var)3*–*7* homozygous mutant chamber is fragmented.
(TIF)

Figure S3 (**A**) Quantitative RT-PCR analysis on the indicated transposons in *Su(var)3*–*7^R2a8^*/*Su(var)3*–*7^14^* mutant ovaries (grey bars) and female carcasses (black bars). Histograms represent the fold changes in RNA levels relative to *Su(var)3*–*7^R2a8^*/*TM6* siblings (n = 3; * : p<0,05; ** : p<0,01). (**B**) Absence of *Su(var)3*–*7^R2a8^* maternal effect on transposon activity in ovary. We compared by qRT-PCR the level of transposon expression in ovaries from *Su(var)3*–*7^R2a8^*/*TM6* females issued either from homozygous or heterozygous *Su(var)3*–*7^R2a8^* mothers. Shown are the fold changes in RNA levels of the indicated transposons relative to *Su(var)3*–*7^R2a8^*/*TM6* females issued from heterozygous mothers (n = 3). (**C**) *Su(var)3*–*7* regulates piRNA *cluster1*/*42AB* transcription. Quantitative strand-specific RT-PCR analysis of c*luster1* from *w^1118^* control and *Su(var)3*–*7^R2a8^* mutant ovaries. Shown are the fold changes in RNA levels from sense (black) and antisense (red) transcripts relative to the control (n = 3; * : p<0,05). The location of the PCR primers is shown in Figure 3C.
(TIF)

Figure S4 **Loss of** ***Su(var)3***–***7*** **faintly reduces ovarian piRNA content.** (**A**) Annotation of small RNA (19–29 nt) libraries of heterozygous and homozygous *Su(var)3*–*7^R2a8^* ovaries. The amount of small RNA categories is indicated as percentage of the total number of reads that matched the *D. melanogaster* genome 5.47. Normalization factors used for library comparisons are indicated. (**B**) Length profile of normalized 23–29 nt small RNAs (grey antisense, black sense). Sense and antisense piRNAs are reduced by approximately 20%, with a marked reduction of the 26–27 nt RNAs in the homozygous mutant ovaries.
(TIF)

Figure S5 ***Su(var)3***–***7*** **mutation does not modify Piwi localization and protein level in ovary.** (**A**–**P**) Confocal sections crossing through the germline (**A**–**D**, **I**–**L**) and the follicular epithelium (**E**–**H**, **M**–**P**) of control (upper panel) and *Su(var)3*–*7* mutant (lower panel) egg chamber. Ovaries were stained with anti-Piwi (red) and anti-Su(var)3–7 (green), DNA was labeled with DAPI (blue). (**Q**) Western blot of Piwi in control (*w^1118^*, lane 1), *Su(var)3*–*7^R2a8^*/*TM6* (lane 2) and *Su(var)3*–*7^R2a8^* homozygote mutant (lane 3) ovaries. Tubulin was used as a loading control.
(TIF)

Table S1 **Cytological location of HP1, Su(var)3**–**9 and Su(var)3**–**7 on** ***otu^11^*** **pseudonurse cell polytene chromosomes.** The relative levels of anti-HP1, anti-Su(var)3–9 and anti-Su(var)3–7 staining at each locus were estimated by eye: (+++) high; (++) moderate; (+) weak; (±) very weak staining.
(PDF)

Table S2 **List of oligonucleotides used for quantitative RT-PCR.**
(PDF)

File S1 **Supporting materials and methods.**
(DOCX)
