# Supplementary figures and images for: The Drosophila Su(var)3–7 Gene Is Required for Oogenesis and Female Fertility, Genetically Interacts with piwi and aubergine, but Impacts Only Weakly Transposon Silencing
Source: PLoS One. 2014 May 12;9(5):e96802. doi: 10.1371/journal.pone.0096802 (PMC4018442; doi:10.1371/journal.pone.0096802)

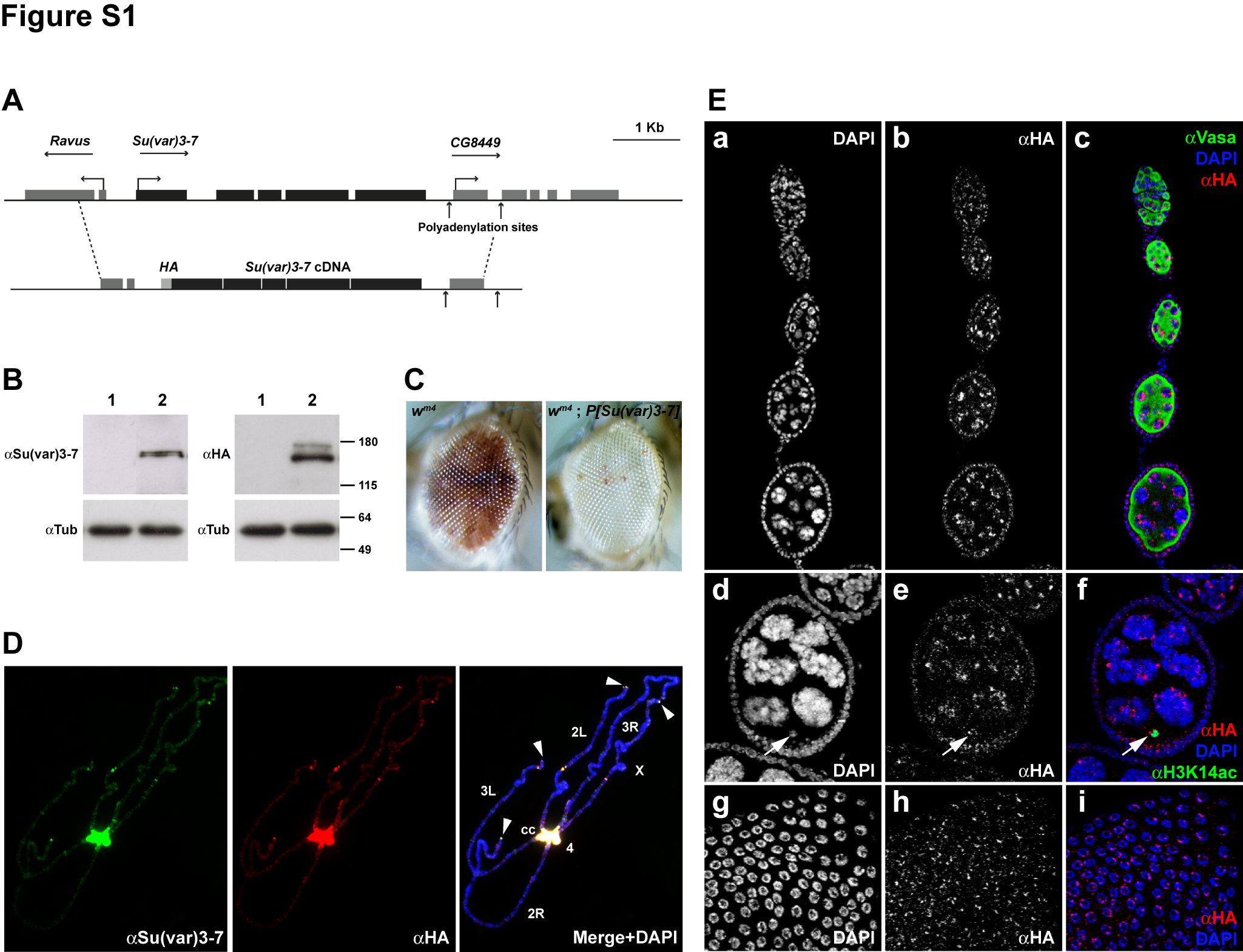

Supplement: Figure S1 — Characterization of the P [ HA:Su(var)3–7 ] transgene. (A) Schematic representation of the HA:Su(var)3–7 construct and of the Su(var)3–7 endogenous locus. (B) Western blotting on crude extracts from S2 cells transfected with (1) empty vector and (2) plasmid encoding HA:Su(var)3–7. Membranes were probed with either anti-HA or anti-Su(var)3–7, anti-tubulin was used as a loading control. HA-tagged Su(var)3–7 migrates as a doublet at 170 kDa as previously observed with the endogenous protein [1]. (C) P[HA:Su(var)3–7] acts as an enhancer of variegation. Adult eyes of wm4/+ and wm4/+; P[HA:Su(var)3–7] flies. (D) Immunostaining on polytene chromosomes from salivary glands of yw; P[HA:Su(var)3–7] third instar larvae stained with anti-Su(var)3–7 (green) and anti-HA (red) antibodies, DNA was visualized by DAPI (blue) staining. Endogenous and HA-tagged Su(var)3–7 proteins have similar binding pattern and localize at the chromocenter (cc), on telomeres (arrowheads) and on several euchromatic sites scattered along the chromosome arms. (E) In ovaries, the P[HA:Su(var)3–7] transgene localizes in somatic and germline cells similarly to endogenous protein. (a–i) Confocal images of P[HA:Su(var)3–7] expressing ovary stained with anti-HA (red) and anti-Vasa (c, green) or anti-H3K14ac (f, green) antibodies, DNA was visualized by DAPI (blue) staining. Anti-Vasa was used as germline cell marker and anti-H3K14ac to label the karyosome. (TIF) [file pone.0096802.s001.tif]

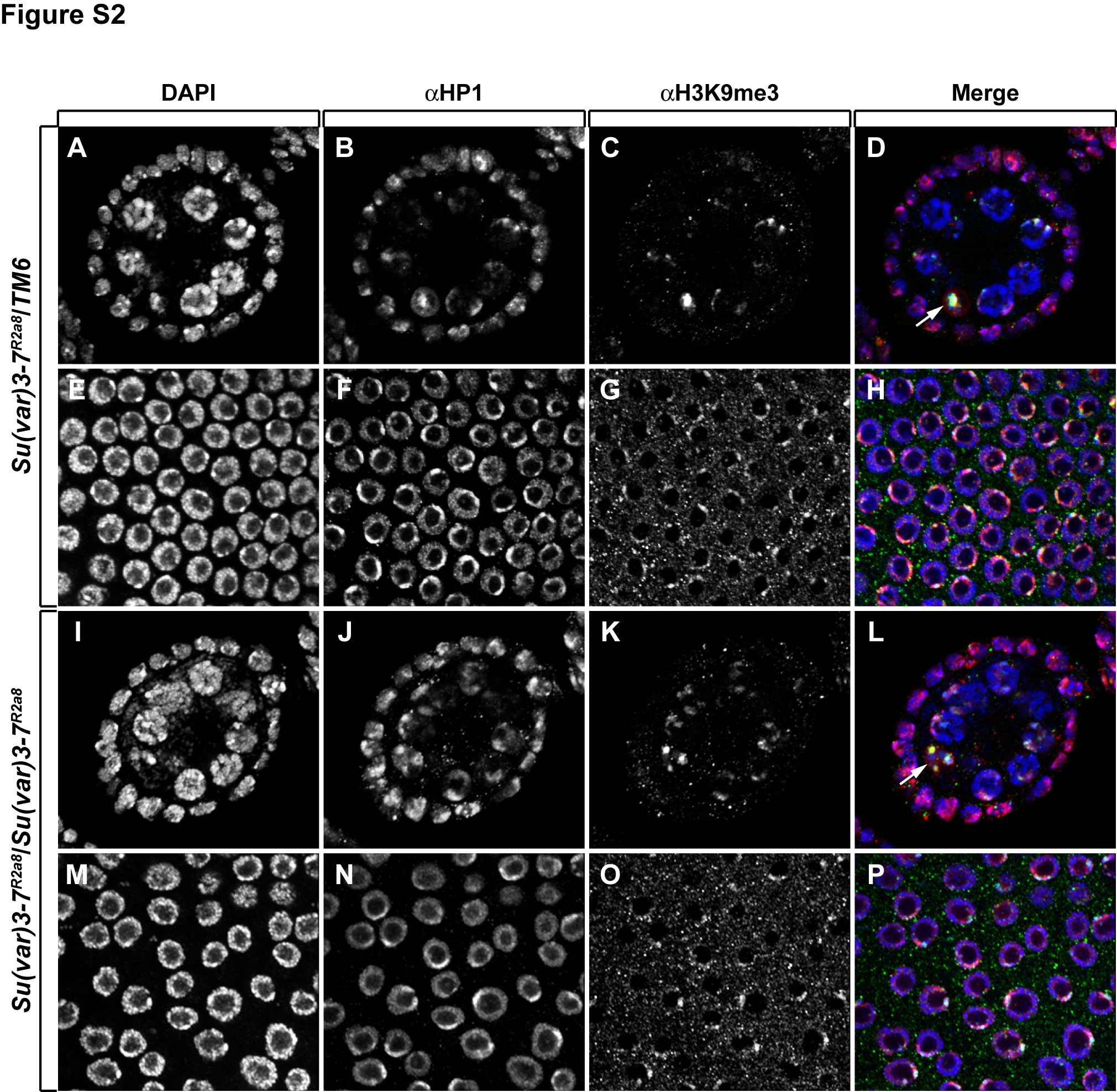

Supplement: Figure S2 — Su(var)3–7 mutation does not impair HP1 and H3K9me3 patterns in ovaries. (A–P) Confocal sections crossing through the germline (A–D, I–L) and the follicular epithelium (E–H, M–P) of control (upper panel) and Su(var)3–7R2a8 homozygous mutant (lower panel) egg chamber. Ovaries were stained with anti-HP1 (red) and anti-H3K9me3 (green), DNA was labeled with DAPI (blue). HP1 and H3K9me3 are localized mainly in heterochromatin territories of nurse cells and somatic follicular cells as well as in the karyosome (arrow); the oocyte nucleus from the Su(var)3–7 homozygous mutant chamber is fragmented. (TIF) [file pone.0096802.s002.tif]

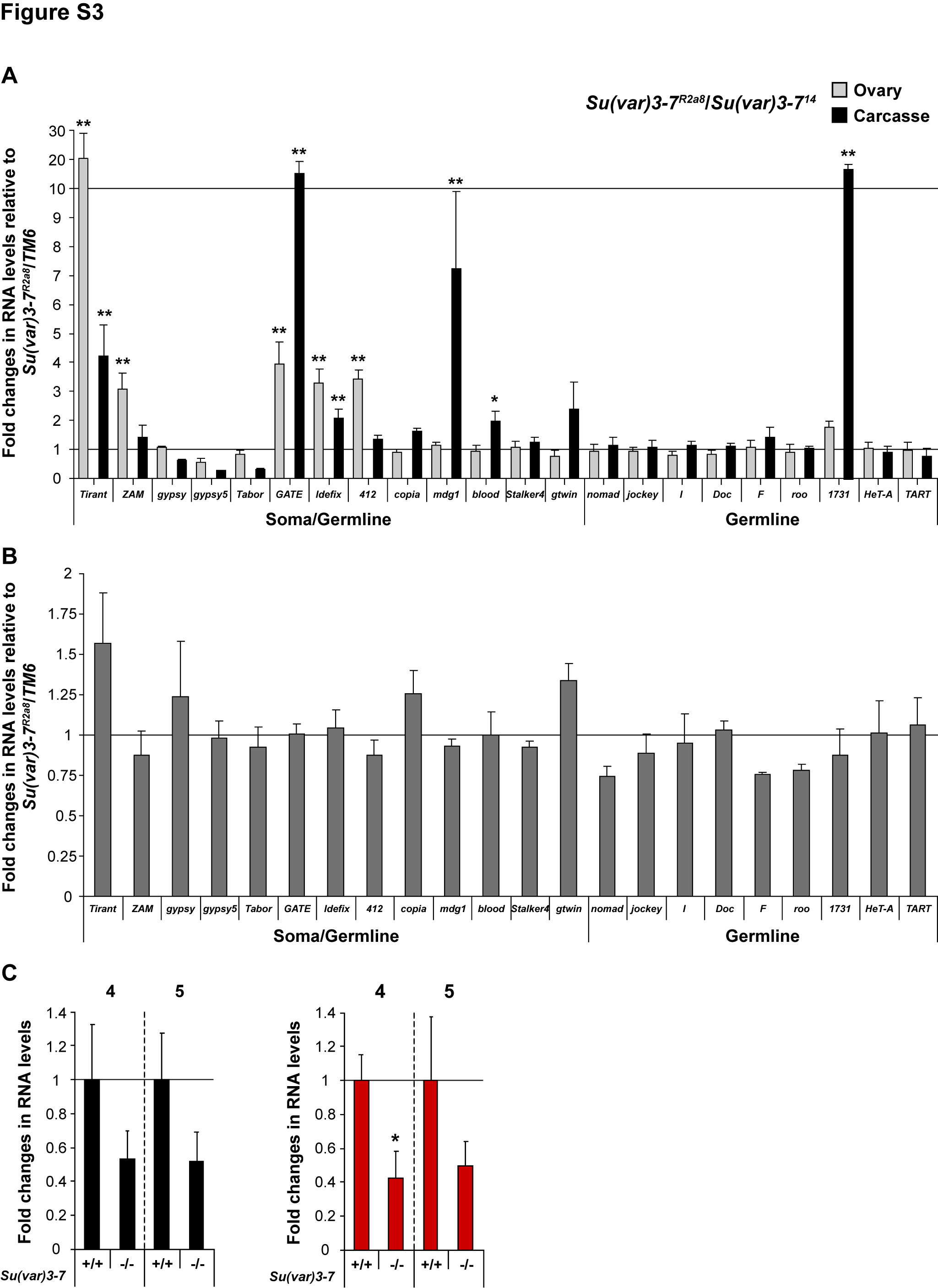

Supplement: Figure S3 — (A) Quantitative RT-PCR analysis on the indicated transposons in Su(var)3–7R2a8/Su(var)3–714 mutant ovaries (grey bars) and female carcasses (black bars). Histograms represent the fold changes in RNA levels relative to Su(var)3–7R2a8/TM6 siblings (n = 3; * : p<0,05; ** : p<0,01). (B) Absence of Su(var)3–7R2a8 maternal effect on transposon activity in ovary. We compared by qRT-PCR the level of transposon expression in ovaries from Su(var)3–7R2a8/TM6 females issued either from homozygous or heterozygous Su(var)3–7R2a8 mothers. Shown are the fold changes in RNA levels of the indicated transposons relative to Su(var)3–7R2a8/TM6 females issued from heterozygous mothers (n = 3). (C) Su(var)3–7 regulates piRNA cluster1/42AB transcription. Quantitative strand-specific RT-PCR analysis of cluster1 from w1118 control and Su(var)3–7R2a8 mutant ovaries. Shown are the fold changes in RNA levels from sense (black) and antisense (red) transcripts relative to the control (n = 3; * : p<0,05). The location of the PCR primers is shown in Figure 3C. (TIF) [file pone.0096802.s003.tif]

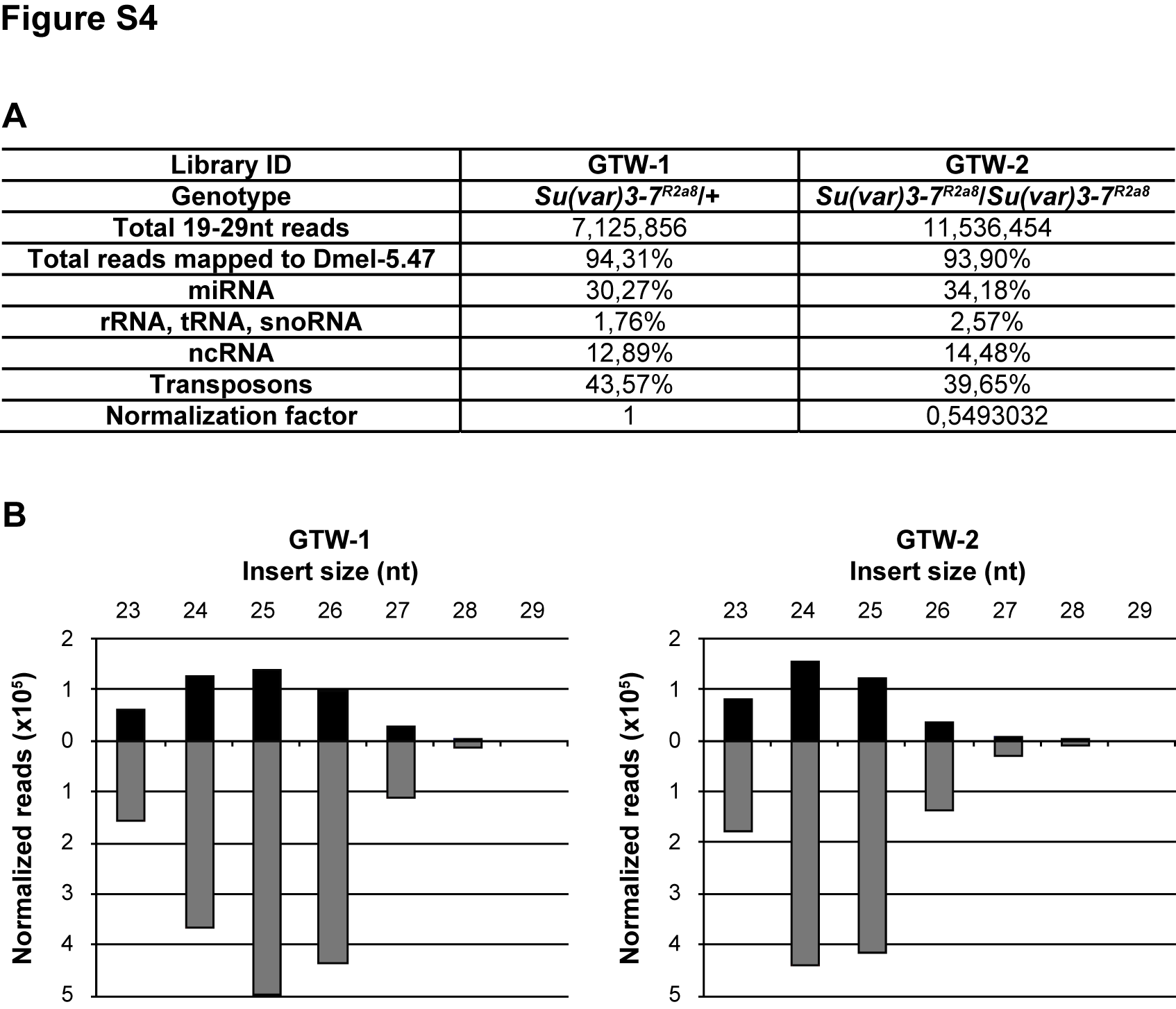

Supplement: Figure S4 — Loss of Su(var)3–7 faintly reduces ovarian piRNA content. (A) Annotation of small RNA (19–29 nt) libraries of heterozygous and homozygous Su(var)3–7R2a8 ovaries. The amount of small RNA categories is indicated as percentage of the total number of reads that matched the D. melanogaster genome 5.47. Normalization factors used for library comparisons are indicated. (B) Length profile of normalized 23–29 nt small RNAs (grey antisense, black sense). Sense and antisense piRNAs are reduced by approximately 20%, with a marked reduction of the 26–27 nt RNAs in the homozygous mutant ovaries. (TIF) [file pone.0096802.s004.tif]

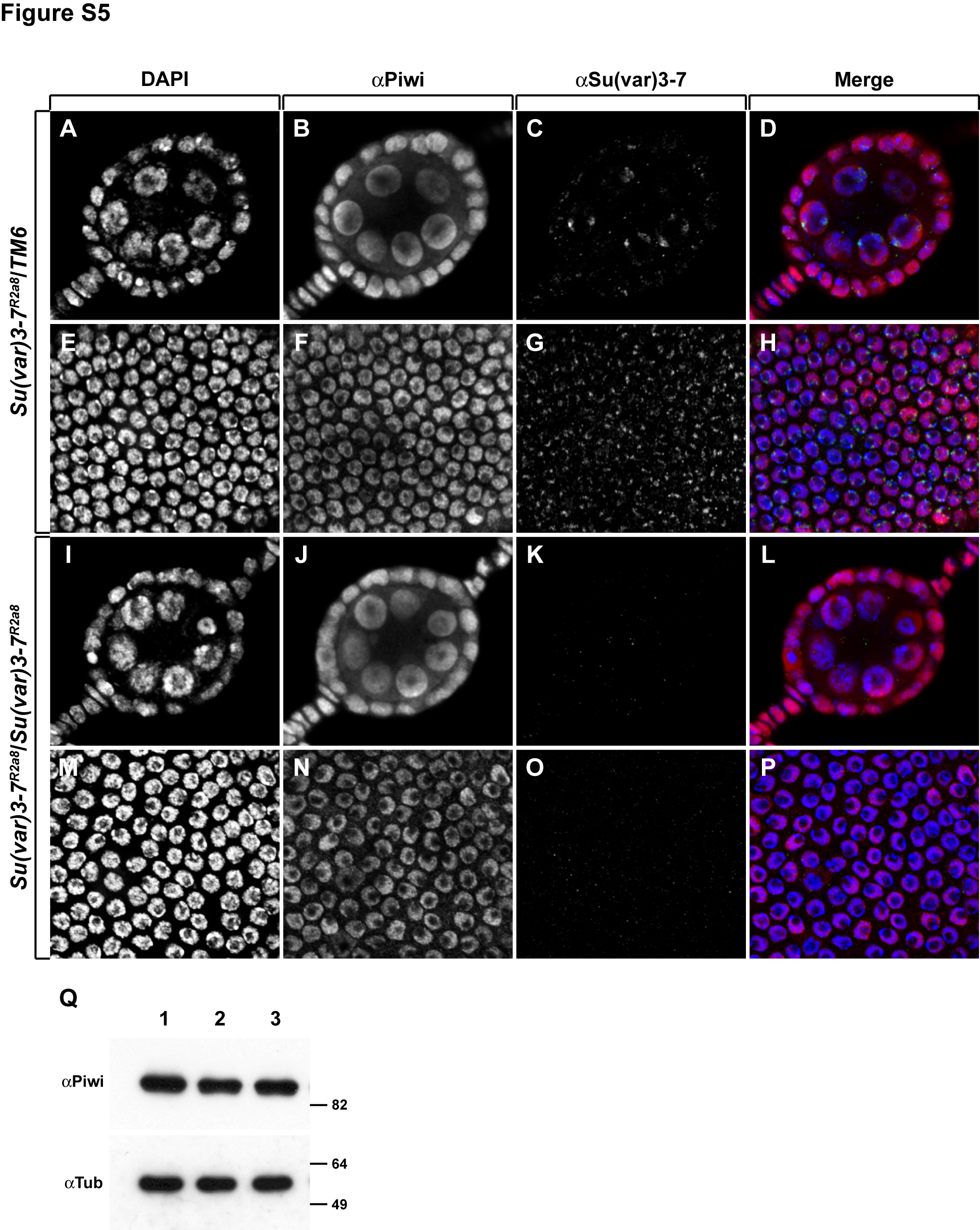

Supplement: Figure S5 — Su(var)3–7 mutation does not modify Piwi localization and protein level in ovary. (A–P) Confocal sections crossing through the germline (A–D, I–L) and the follicular epithelium (E–H, M–P) of control (upper panel) and Su(var)3–7 mutant (lower panel) egg chamber. Ovaries were stained with anti-Piwi (red) and anti-Su(var)3–7 (green), DNA was labeled with DAPI (blue). (Q) Western blot of Piwi in control (w1118, lane 1), Su(var)3–7R2a8/TM6 (lane 2) and Su(var)3–7R2a8 homozygote mutant (lane 3) ovaries. Tubulin was used as a loading control. (TIF) [file pone.0096802.s005.tif]
