# Supplementary material for: The Drosophila Su(var)3–7 Gene Is Required for Oogenesis and Female Fertility, Genetically Interacts with piwi and aubergine, but Impacts Only Weakly Transposon Silencing
Source: PLoS One. 2014 May 12;9(5):e96802. doi: 10.1371/journal.pone.0096802 (PMC4018442; doi:10.1371/journal.pone.0096802)
Supplement: Table S1 — Cytological location of HP1, Su(var)3–9 and Su(var)3–7 on otu11 pseudonurse cell polytene chromosomes. The relative levels of anti-HP1, anti-Su(var)3–9 and anti-Su(var)3–7 staining at each locus were estimated by eye: (+++) high; (++) moderate; (+) weak; (±) very weak staining. (PDF) [file pone.0096802.s006.pdf]

Table S1 : Cytological location of HP1, Su(var)3-9 and Su(var)3-7 on *otu*^{11} pseudonurse cells' polytene chromosomes.

X	HP1	Su(var)3-9	Su(var)3-7	2L	HP1	Su(var)3-9	Su(var)3-7	2R	HP1	Su(var)3-9	Su(var)3-7
1A1	**	*		21A	***	***	*	41	***	***	***
1A5-6		*		21B	***	***	*	42B	***	**	*
1B	±			21C	*	±		43A	**	**	
1C	±			21D	±			43B	±	±	
1D	±			21E	±		*	43D	*	±	
1E	±			22A4-5		±		44A	±		
1F1-2	±	*		22B	±	±		44C	±		
1F3	±			22C			±	44D	±	±	
2B3-4	±			22E	±			44F		±	
2B7-8	±			22F	±	±		45A	*	±	
2D	±	±	*	23A		±	*	45D	*	**	
2E	±	±		23B	**	*		45E		*	*
2F				23C	*			45F	*		*
3A1-2	**	*	**	23E	**	*		46C	±	±	*
3A3-4	*	*	**	23F1-2			±	46F	*	*	
3C1	**	±		23F3-4	±	*		47A	*	±	±
3C2-6	***	**		24A	±			47D1-2	±	±	*
3C9-10	*	±		24B	±	±		47D5-6			±
3D		±	**	24EF	***	*		47E	*	*	
3E	*			25B1-2		*		47F	*	*	
3F1-2	*	±		25B4-5	*		*	48A	±	±	±
3F4-5	*	±		25C	±	±	*	48D	±	±	
4B	*	±		25D	**	*	**	48E10-11	**	***	
4C3-4	*	*		25E	±	±	**	49C	±		
4C7-8	*		*	25F-26A	±	±		49F	*	*	*
4E	±			26B			**	50A	*	*	
4F1-4	*	**	*	26C	*	*		50B		*	
4F13-14	**	**		26E	±	±		50C1-4	*	*	
5A	*	±	±	26F		*		50C9-10	±		
5B			±	27F	*		*	50D	***	***	
5C	±			28A1-2	*	*		50E	**	**	*
5D	±	*		28A6	*			50F	±		
5F	±	*		28B	*			51A6-8	**	***	
6A	*	*	*	28C1-2	±		±	51B4-5			±
6B	***			28C?-?			±	51B7-8	**	*	±
6C4	*			28F	±		±	51B10	*		
6C6-7	**			29D			±	51C			*
6D	***	*		29E	*	±		52B	±		
6E	±		**	29F	*	±	*	52D	*		
7B	**	*		30A		±	±	52E		*	
7C	±		±	30D	±		*	52F9	±	±	
7D1-2	±	±		30E			*	53A	***	***	
7D3			±	31A	*	*		53B		±	
7D19-22	*	±		31CD	*	±		53E	**	*	
7E	*	*		32A	±	**		54A	±	*	
8A	±			32B		±		54C	*		
8C	**	*	*	32C	*	**		54D	*		
8D4-5	±			33A			±	54F	*	*	
8D8-9	±			33B1-2	**	*		55A	*	*	
8E	±	±		33B?-?			**	55C	±	±	
9A	±		±	33D	*			56A	±	*	
9B1-2	*			33F			±	56D	±		

**9B5-6**	*		
**9E**	*	*	
**9F**			±
**10B15-16**		*	*
**10C1-2**	±	±	*
**10C7-8**	±		
**10D**	±	±	
**10E**	±	±	
**10F**	±		
**11A1-2**	*		±
**11A6-9**	*	*	
**11B1-2**	**		*
**11B7-8**	*		
**11C**	±	*	
**11D1-2**	±	±	
**11D5-6**	±	**	
**11D8**	**		***
**11E**	±		
**11F**	**	*	*
**12A**	*		
**12B**	**	*	*
**12C**	*		
**12E1-2**	*	*	
**12E6-7**	**	*	**
**13A**	±		
**13B**	*	*	**
**13C**	*	±	
**13D**	*	±	*
**13E5-6**	*	*	*
**14A**	**	**	**
**14B**		**	
**14D**	**	**	±
**15A**		±	*
**15C**		±	**
**16D**		±	
**16F**	**	±	
**17A**	*		
**17B**	*	*	**
**17C**	**	**	**
**17D**	*		
**17E**	*	*	
**18A**	**	*	
**18B**	±		
**18C**	**	±	
**18D**	±		
**18E**	±		
**18F**	±		
**19A**	*		
**19B**	**		±
**19C**	**		
**19D**		**	
**19E**	**	*	
**20**	***	***	*

**34B**	*	*	
**34C**	*	*	
**34D**			±
**34E**	±	*	
**35C**			*
**35E**			*
**36A1-2**	±		±
**36A4-5**	*	*	
**36A6-7**	*	*	
**36B**	*		
**36C**	*		
**36D**	*		
**36E**	*	±	±
**36F**	*	*	
**37A4-5**	**		
**37B1-2**		**	
**37B7-8**	±	±	
**37C**			±
**37E**	*	±	
**38A**	**	**	
**38B**	**	**	*
**38CD**	*	±	
**39A**	*		
**39DE**	***	***	**
**40BF**	***	***	***

**56E**	±		**
**57B**	*	±	
**57D**	**	**	
**58D**	±		
**58E**	±		±
**58F**	*		
**59A**	*	*	
**59B**	**		
**59C**		*	
**59D**	*		
**59F**	±		±
**60C**	±		±
**60D**	***	***	
**60E**	±		*
**60F**	***	*	*

3L	HP1	Su(var)3-9	Su(var)3-7
61A	*		
61B	*		
61C	±		
61E	*		
61F	±	*	
62A	±		
62B	**		
62C	**	**	
62D	**		*
62E	±		
62F	±		
63A	±	±	
63B1-2			**
63B14	**		±
63D			±
63E1-2	*		
64A	**	*	
64B	*		
64C8	±		
64C11-12		±	
64C14	*	±	
64D1-2	*		
64E	±		
65A	±		
65C	±		
65F	*	±	
66B1-2	***		
66B3-4		*	
66C	±		
66D1-2	*	*	
66D10-11	±		
67A	*	*	
67B?-?			**
67B10-11	*	*	
67C7-8			**
67D1-2	±		**
67E	±		
67F	*	*	
68A	*	*	*
68C	**	**	**
68D	*	±	±
68E	**	±	
69A	**	*	*
69B			*
69D	*	*	
69E	*	±	±
69F1-2	*	±	
69F5	*		
70A	*	*	
70C	*	*	
70D	*		
70E1-2	*	*	*
70E 7	**		
71A	±	*	*
71D		±	

3R	HP1	Su(var)3-9	Su(var)3-7
81F	***	***	***
82B	±		
82C	*	±	
82D1	±		
82E	±		
83A	±		
83B	*		
83C	±		
83F	*		
84A			*
84B		±	
84D1-4		*	
84D9-12	**		
84E10-11	**	*	*
84F1-2	**	*	*
85B	±		
85C	±		
85D19	±	±	
85D21-22	±	±	
85F	***	*	**
86C	*	*	
86D	*	*	
86E1-2	±		
86E7-8	±		
87A	*	*	
87B	*		
87C	***	***	*
87D	**	*	
87E	*		
88A		±	
88B		±	
88C		**	
88D	**		**
88E9-11		±	
88F	±		
89A1-2			±
89A8-9	±	*	
89B12-13			±
89C	*		
89D	*	*	±
89E	±	**	
90B	±		
90C	±		±
90E	±	*	*
90F	±		
91D	*	*	
91F1-2	*	±	±
91F10-11	*	±	
92A	*	±	
92C	**	*	
92D			**
93A	**	*	
93B	*	*	*
93D	±	*	
93E 1	±	±	***

Chr 4	HP1	Su(var)3-9	Su(var)3-7
101	***	***	***
102	***	±	*

**71F**	±	±	*
**72A**	**	*	
**72C**			*
**72E1-2**		±	±
**72E 4**	*		
**72F**			±
**73A**		±	
**73B**	*		*
**73C**		**	
**73E**			±
**74F**			±
**75A1-2**	*	*	±
**75A4-5**	**	**	*
**75B1-2**	**	*	
**75B4**	**		
**75E**	**		
**75F1-2**		*	
**75F?-?**			*
**76A**	±		
**76B**	±		
**76D**	*		
**77B**	**		
**77F**	±		
**78A**	±	*	
**78D**	*	*	
**78E**	*		
**78F**	*		
**79B**	*	*	*
**80**	***	***	***

**93E9-10**	**	±	±
**93F1-2**			**
**93F?-?**			**
**94A**	*	±	±
**94C**			±
**94D**	*	±	±
**94F**			*
**96E**	**	*	
**96F**	*	*	±
**97B**			**
**97C2**	±		
**97C5**	*		
**98A11-12**	±	±	*
**98E**	*	*	
**98F1-2**	**	*	
**98F?-?**			**
**99A**			*
**99B9-10**	*		
**99E 1**	*		
**99E 4**	*		**
**99F**	**		
**100A1-2**		±	**
**100B**		±	
**100C**	*		
**100EF**	**	*	
