# Supplementary material for: The Drosophila Su(var)3–7 Gene Is Required for Oogenesis and Female Fertility, Genetically Interacts with piwi and aubergine, but Impacts Only Weakly Transposon Silencing
Source: PLoS One. 2014 May 12;9(5):e96802. doi: 10.1371/journal.pone.0096802 (PMC4018442; doi:10.1371/journal.pone.0096802)
Supplement: Table S2 — List of oligonucleotides used for quantitative RT-PCR. (PDF) [file pone.0096802.s007.pdf]

Table S2

Control genes

Gene name	Primer forward	Primer reverse
α tubulin	TTTTCCTTGTCGCGTGTGAA	CCAGCCTGACCAACATGGAT
Ef1g	GTGTTCATGTCGTGCAATCTCA	CGCCTTGCGCATCTTGT
Gapdh1	ATTTCGCTGAACGATAAGTTCGT	CGATGACGCGGTTGGAGTA
rp49	GCGCACCAAGCACTTCATC	TTGGGCTTGCGCCATT

Transposable elements

TE name	Primer forward	Primer reverse
1731	TGTCGTCGCGAGAAAGTAAACA	CACTATCCAGCGCATTTAACAAA
412	GCGAAGGAATCTGGAGTCCC	TCGATGGTCGCTCACAAGTC
blood	AGCGCTCACCATCATCAAACT	AGTTCGGTGGCTTCTTTCTATTGT
copia	TCATGTGAAATTTGTGAACCCTG	AGGAAGTCTTGCCTGTTTACCATT
Doc	ACGACAGACCGCGTTCTTG	TCTCTTCGCTCCACGACTTGT
F-element	GAGGTCAATCTGGTTGGTGGTT	TGATCACTCGCCTGTACTAATTCAC
GATE	CGACTTAAAGCACATGCGACAA	CGGCGTGATGTGGCATATAA
gtwin	TGACTATGTGTCATGGCGGC	TTGAAGATCTCGTATGCGTCCA
gypsy	CAAGGATTGGAAATGGTTAGGC	TCGTAGTATTGCATTAGTGGTAGGTCTC
gypsy5	AACCGGAACAACAGTGGATTG	CAGCCCTTAGTTGATTCTTGCTG
HeT-A	GGCAGCATATTAGCGCGTACA	TTTGCCGCCAGCTTTTGT
I-element	AATACGGCATACTGCCCCC	CGATTGTTTCGGAGGTGAGG
Idefix	CTACTACGATGGCGCACCTG	TCCACTTGGTTGACGAATCCA
jockey	ACGACTCAATCTAGGGCTCGTG	CGTCCATTCTCGTATTGATGG
mdg1	CGCCAGCAACAGCATTTG	GCATACTCATTTCCGTTTTCTGATC
nomad	GATTCGCAGGCCCATATCAA	GCCTGAACCAGGAGAAAAAGG
roo	GTCTGAGGCATCCGTTTGGT	GTCGAACAAAGCTGCTCGCT
springer	CGGCTTACCCGAAATCGAA	TTGACCCTTTGCCTTTTTGG
Stalker4	AATCAAGCGGCGCTGAGT	CGCTTCCTTCTAGCGTTTCAAT
Tabor	GGGTTGGTTCGGATCTGACG	ACGTTGTTCACGACATTAGCCG
TART	GCCATCTCAAGGTCCTGCTACTAA	TTCGATACCCGTTTCCTTTCA
Tirant	TCGGAAGAACCCAAATCAATATC	GGCGGGCAGAATCGTTT
X-element	GGAAAACCCTTCGGCTACAGA	AGCTGTGAGATGCGCAAGTAAA
ZAM	AAAGCACCCCCTTACCGC	CGTTGCCCGAATCCATATTT

piRNA clusters

Cluster name	Primer forward	Primer reverse
***Cluster 2* (1)** (Klattenhoff et al. 2009)	CTGCTTTGTGCTTGGAGATG	TCTGCACAGATTCTGAAATTGAA
***flamenco* (2)** (Klattenhoff et al. 2011)	TGAGGAATGAATCGCTTTGAA	TGGTGAAATACCAAAGTCTTGGGTCAAC
***flamenco* (3)** (Haase et al. 2010)	CGGCAGTTTTCTGCAGTGTA	TCAAGCCCTCCAACGTAATC
***Cluster 1* (4)** (Klattenhoff et al. 2009)	CGTCCCAGCCTACCTAGTCA	ACTTCCCGGTGAAGACTCCT
***Cluster 1* (5)** (Klattenhoff et al. 2009)	GTGGAGTTTGGTGCAGAAGC	AGCCGTGCTTTATGCTTTAC
**Cluster name**	**RT-PCR Primer (plus strand)**	**RT-PCR Primer (minus strand)**
***Cluster 1* (4)** (Klattenhoff et al. 2009)	CGAAGCCTTAGATCTCGCTCC	ACATCAGGAACACAGCGAGGTG
***Cluster 1* (5)** (Klattenhoff et al. 2009)	GGTGCAAATGTCTCATCATAATCAGTC	GATGAAATTGAATTTCGTGATGACAGATC
