# Supplementary material for: The Drosophila Su(var)3–7 Gene Is Required for Oogenesis and Female Fertility, Genetically Interacts with piwi and aubergine, but Impacts Only Weakly Transposon Silencing
Source: PLoS One. 2014 May 12;9(5):e96802. doi: 10.1371/journal.pone.0096802 (PMC4018442; doi:10.1371/journal.pone.0096802)
Supplement: File S1 — Supporting materials and methods. (DOCX) [file pone.0096802.s008.docx]

Supporting MATERIALS AND METHODS:

**Construction of the *P[HA:Su(var)3-7]* transgene**:

The *Su(var)3-7* cDNA and upstream promoter sequences were recovered from the expression plasmids pNB40 [[2](#_ENREF_2)], and subcloned into the pBluescript (KS) vector (Stratagene). The resulting plasmid pKS-*Su(var)3-7^Lop^* was modified by insertion of a single hemagglutinin (HA) epitope tag in frame at the 5’ end of the coding sequence (Figure S1). The construct was then verified by sequencing and cloned into the *P-element* transformation vector pC4-yellow [[3](#_ENREF_3)].

**Immunostaining of polytene chromosomes:**

Salivary glands from third-instar larvae were dissected and squashed as described previously [[4](#_ENREF_4)]. Briefly, salivary glands were dissected in Cohen's buffer, fixed for 2 minutes in 2% formaldehyde, 2% Triton X-100 and then squashed in 2% formaldehyde, 45% acetic acid. Primary antibodies were used at the following dilutions: rabbit anti-Su(var)3-7 [[1](#_ENREF_1)], 1:10 ; mouse anti-HA (16B12, Covance), 1:200. As secondary antibodies we used a Cy3-conjugated goat anti-mouse (Jackson Laboratories), 1:400 and a DTAF-conjugated goat anti-rabbit (Jackson Laboratories), 1:200. DNA was counterstained for 10 minutes with 0.1 μg/ml DAPI.

**Cell culture and western blotting:**

Schneider S2 cells were transiently transfected with recombinant plasmid DNA using Fugene6 reagent (Roche). All transfections were balanced for a total of 0.5 μg/ml of DNA with empty vector derivative from the pMT/V5-His (Invitrogen). Expression of the pMT vectors were induced for 2 to 3 days by adding CuSO_4_ at 500 μM.

Crude extracts were electrophoresed through 8% to 10% SDS-polyacrylamide gels, and electrotransferred to PVDF membranes (Millipore). Membranes were probed with primary antibodies used at the following dilutions : rabbit anti-Su(var)3-7 [[1](#_ENREF_1)], 1:1000 ; mouse anti-HA (16B12, Covance), 1:2000 ; mouse anti-tubulin (DM1A, Sigma), 1:2000 ; mouse anti-Piwi (P4D2, kindly provided by K. Saito), 1:500. Proteins were detected by chemiluminescence using an HRP-conjugated goat anti-mouse or anti-rabbit secondary antibody (Bio-Rad) at 1:10000, and an HRP chemiluminescent substrate (SuperSignal West Pico from Pierce or the Lumi-Light^PLUS^ Western Blotting Substrate from Roche).

**Strand specific RT-PCR:**

Strand specific RT-PCR analysis was performed as described in [[5](#_ENREF_5)]. Briefly, RNA samples were extracted from whole ovaries with Trizol followed by DNase treatment. Plus or minus strand primers to the indicated regions (Table S2) were mixed and strand-specific cDNA were synthesized using the SuperScript III RT (Invitrogen) enzyme following the manufacture’s protocol. The resulting cDNAs were then used as template for quantitative RT-PCRs as depicted previously (see Materials and Methods).

Supporting REFERENCES:

1. Cléard F, Delattre M, Spierer P (1997) SU(VAR)3-7, a Drosophila heterochromatin-associated protein and companion of HP1 in the genomic silencing of position-effect variegation. Embo J 16: 5280-5288.

2. Cléard F, Matsarskaia M, Spierer P (1995) The modifier of position-effect variegation Suvar(3)7 of Drosophila: there are two alternative transcripts and seven scattered zinc fingers, each preceded by a tryptophan box. Nucleic Acids Res 23: 796-802.

3. Sigrist CJ, Pirrotta V (1997) Chromatin insulator elements block the silencing of a target gene by the Drosophila polycomb response element (PRE) but allow trans interactions between PREs on different chromosomes. Genetics 147: 209-221.

4. Spierer A, Seum C, Delattre M, Spierer P (2005) Loss of the modifiers of variegation Su(var)3-7 or HP1 impacts male X polytene chromosome morphology and dosage compensation. J Cell Sci 118: 5047-5057.

5. Klattenhoff C, Xi H, Li C, Lee S, Xu J, et al. (2009) The Drosophila HP1 homolog Rhino is required for transposon silencing and piRNA production by dual-strand clusters. Cell 138: 1137-1149.
